# Activation of an adaptive antitumor immune response by the polymeric fluoropyrimidine CF10 involves TS/Top1 dual targeting

**DOI:** 10.1016/j.biopha.2026.119399

**Published:** 2026-04-21

**Authors:** Akanksha Behl, Taylor M. Young, Xue Ma, Edward Cedrone, Marina A. Dobrovolskaia, John F. Whitesides, William H. Gmeiner

**Affiliations:** a Department of Cancer Biology, Wake Forest University School of Medicine, Winston-Salem, Wake Forest University School of Medicine, Winston-Salem, NC 27157, USA; b Department of Orthopedic Surgery and Rehabilitation, Medical Center Boulevard, Winston-Salem, NC 27157, USA; c Nanotechnology Characterization Laboratory, Cancer Research Technology Program, Frederick National Laboratory for Cancer Research sponsored by the National Cancer Institute, Frederick, MD 21701, USA; d Microbiology & Immunology, Wake Forest University School of Medicine, Winston-Salem, NC 27157, USA

**Keywords:** Immunogenic cell death (ICD), Dendritic cell maturation, CF10, 5-fluorouracil (5-FU), Colorectal cancer, Antitumor immunity

## Abstract

5-Fluorouracil (5-FU)-based regimens remain the backbone of therapy for metastatic colorectal cancer (mCRC), yet durable responses are rare.CF10, a next-generation polymeric fluoropyrimidine, has demonstrated superior antitumor activity compared with 5-FU in preclinical models. Here, we evaluated whether CF10 more effectively induces immunogenic cell death (ICD) and promotes antitumor immunity, while characterizing its dual mechanism involving thymidylate synthase (TS) inhibition and replication stress through Topoisomerase 1 cleavage complex (Top1cc) stabilization and γ-H2AX accumulation. In murine (MC38) and human (HCT116) colorectal cancer cells, CF10 induced significantly higher levels of ICD markers—extracellular ATP, HMGB1 release, and surface calreticulin—than 5-FU and triggered robust Top1cc stabilization with γ-H2AX foci formation. Conditioned media from CF10-treated cells enhanced dendritic cell (DC) maturation and secretion of TNF-α, IL-1β, CCL2, and CCL4. In vivo, CF10 treatment in C57BL/6 mice bearing orthotopic MC38 liver metastases increased CD4^+^, CD8^+^, and γδ T-cell infiltration, reduced myeloid-derived suppressor cells (MDSCs), and decreased hepatic tumor burden. CF10 also decreased FoxP3^+^ regulatory T cells and CD206^+^ immunosuppressive macrophages. DC vaccination using CF10-conditioned supernatants modestly extended survival and increased tumor-infiltrating T cells compared with 5-FU or untreated controls. CF10 elicits a dual-action mechanism—cytotoxicity via TS inhibition and Top1cc-dependent replication stress (γ-H2AX) and immunogenicity through ICD induction—driving potent DC activation and adaptive immune responses. These findings position CF10 as a promising immunomodulatory chemotherapeutic for mCRC and support clinical evaluation, including combination strategies with immune checkpoint blockade.

## 1. Introduction

Colorectal cancer (CRC) is the third leading cause of cancer-related death, with liver metastases representing the most common and deadly site, occurring in 35–55% of patients during disease progression [1]. Most colorectal cancer (CRC) patients with liver metastases are ineligible for surgical resection, the only potentially curative option. Frontline 5-FU–based regimens, including FOLFOX and FOLFIRI, improve outcomes in metastatic CRC (mCRC), but many patients ultimately require multiple lines of therapy [2]. However, long-term survival is uncommon for mCRC patients with < 14% of patients surviving 5-years post-diagnosis [3]. The central role of cytotoxic chemotherapy in mCRC treatment results from a relative lack of actionable targets for targeted therapy and from mCRC being an “immune-cold” tumor generally non-responsive to immune checkpoint blockade (ICB) therapy [4]. Only patients with MSI-high disease, ~5% of mCRC cases, are generally responsive to ICB therapy. Thus, there remains considerable interest in chemotherapy for mCRC that is immunostimulatory to potentially boost the host antitumor response in a similar manner to for the use of anthracyclines prior to ICB therapy which improves overall survival in breast cancer [5].

The antitumor activity of chemotherapeutic drugs is multifactorial resulting not just from direct cytotoxic effects to cancer cells but also from modulating the tumor microenvironment and facilitating and/or suppressing the host antitumor immune response [6]. 5-FU-based regimens improve outcomes in colorectal cancer (CRC) both as adjuvant chemotherapy reducing risk for disease recurrence [7], and in treatment of metastatic disease improving progression-free and overall survival [8] however the extent that 5-FU efficacy results from a favorable modulation of the antitumor immune in either therapeutic context response is unknown. 5-FU is myeloablative at high concentrations [9] and leukopenia is a frequent and sometimes serious side-effect in mCRC patients treated with 5-FU-based regimens [10]. However, 5-FU also is reported to selectively decrease myeloid-derived suppressor cells (MD-SCs) that contribute to immune tolerance of cancer [11]. The extent to which 5-FU’s generally deleterious effects to immune cells result from thymidylate synthase (TS) inhibition, which is considered its primary mechanism for antitumor activity [12], is also not established however RNA-mediated effects of 5-FU have been implicated in 5-FU-induced leukopenia [13]. Thus, fluoropyrimidine (FP) drugs that more efficiently inhibit TS and cause increased DNA damage relative to 5-FU could modulate the antitumor immune response differently and potentially improve the overall therapeutic response.

Our research focuses on advancing the 2nd-generation fluoropyrimidine (FP) polymer CF10 into clinical development, based on its superior antitumor activity compared with 5-FU in preclinical models [14], including syngeneic, orthotopic CRC liver metastases [15,16]. CF10’s enhanced potency arises from efficient intracellular conversion to FdUMP, the thymidylate synthase (TS)–inhibitory metabolite, and un-expected DNA topoisomerase 1 (Top1) poisoning [14,17,18]. This dual TS/Top1 inhibition increases DNA double-strand breaks and replication stress, which may promote immunogenic cell death (ICD)—a cell death mode marked by release of DAMPs, including extracellular ATP, HMGB1, and surface calreticulin [19]. CRC, especially liver metastases, are typically “immune-cold” with poor cytotoxic T-cell infiltration [4]. Thus, CF10 has potential to induce ICD more effectively than 5-FU, activating dendritic cells and enhancing T-cell–mediated antitumor immunity for more durable therapeutic responses [10].

CF10 induced higher levels of canonical DAMPs (eATP, HMGB1, surface CRT) [19] than 5-FU in human and murine CRC cells, promoting stronger DC maturation (CD80, CD86, MHC-II) [20]. In a syngeneic orthotopic CRC liver metastasis model [21], CF10 enhanced peripheral and intrahepatic T-cell infiltration. DCs pulsed *ex vivo* with CF10-treated supernatants increased CD8^+^ T cells and improved survival, indicating that CF10 potentiates adaptive antitumor immunity through DC-mediated T-cell activation.

## 2. Materials & methods

### 2.1. Reagents

CF10 was purchased from STPharm (Korea) and other chemicals and reagents were obtained from commercial sources (Supplementary Table S1) and used directly without further purification.

### 2.2. CRC cell culture and DAMP secretion

Human (HCT116, LS174T) and murine (MC38) CRC cell lines (ATCC) were cultured under standard conditions [22]. Cells (1 × 10^6^/mL) were treated with CF10 (1 μM), 5-fluorouracil (5-FU, 10 μM), or left untreated (CTR). Supernatants were collected at 0–42 h post-treatment for extracellular ATP (eATP) and HMGB1 quantification using commercial kits (PROMEGA, USA).

### 2.3. Flow cytometry

Plasma membrane calreticulin (CRT) was detected using CRT-specific antibodies. Apoptotic and necrotic populations were assessed by Annexin V-FITC/PI staining (INVITROGEN, USA). Data were acquired on a BD FACSCanto II and analyzed with FlowJo v10.8.1.

### 2.4. Immunofluorescence

CRC cells on coverslips were treated with CF10, 5-FU, Camptothecin (CPT, 1 μM; positive control), or control for 30 h. Cells were fixed, per-meabilized (for γ-H2AX and Top1cc), stained with primary and secondary antibodies, and nuclei counterstained with DAPI. Images were captured on BZ-X700 and FV4000 microscopes and analyzed in ImageJ. Quantification of γ-H2AX and Top1cc foci was performed in ImageJ. Background subtraction was applied using a 50-pixel rolling-ball radius. Nuclei were segmented by Otsu thresholding, and foci were defined as puncta ≥ 0.2–0.3 μm^2^ with fluorescence intensity ≥ 1.5 × the mean nuclear background. CRT fluorescence was quantified by measuring the integrated fluorescence density of each cell, normalized to cell area after background subtraction. For each condition, 8–10 random, non-overlapping fields were selected while avoiding damaged, folded, or heavily clustered regions. All images were acquired using identical exposure and acquisition settings across groups.

### 2.5. Western blot

Protein lysates were prepared in RIPA buffer with inhibitors, resolved by SDS-PAGE, and transferred to nitrocellulose membranes. Antibodies against TS, γ-H2AX, p-eIF2α, and β-actin were used. Bands were visualized by chemiluminescence and quantified by densitometry, normalized to β-actin.

### 2.6. DC cell culture and analysis of maturation markers

Mouse BMDCs were generated from femurs/tibias of C57BL/6 mice; human DCs from PBMCs as described previously [23]. On day 6, DCs were stimulated for 24 h with supernatants from drug-treated CRC cells or LPS (positive control). Surface markers (CD11c, CD80, CD86, MHC-II) were analyzed by flow cytometry.

### 2.7. Cytokine and chemokine production assay and RT-PCR analysis

DC culture supernatants were analyzed for cytokines (IL-6, IL-1β, IL-13, TNF-α) and chemokines (MIP-1α, MIP-1β, RANTES) using cytokine arrays. Cytokine array membranes were analyzed using ImageJ. Spot intensities were measured by defining regions of interest (ROIs) corresponding to each cytokine spot according to the manufacturer’s reference map. Background signal was subtracted from each measurement, and the values were normalized to the positive control spots on the membrane to account for inter-membrane variation. The normalized intensities were then expressed as fold change relative to the control group and visualized as a heatmap to illustrate differential cytokine expression patterns. Total RNA was isolated (RNeasy), reverse-transcribed, and analyzed by qRT-PCR (SYBR Green). GAPDH served as control; relative expression calculated by ΔΔCt method [24,25]. Primer sequences are in Supplementary Table S2.

### 2.8. Cytokine induction in whole blood and PBMCs from human donors

Whole blood from healthy donors was collected under IRB-approved protocol OH99CN046D (NCT00339911) and processed within 2 h for whole blood cultures or PBMC isolation according to the standardized protocol from the NCI’s NCL Assay Cascade described in detail earlier [27]. Samples were incubated with vehicle, CF10, or 5-FU (0.028, 0.14, or 0.71 mg/mL) for 24 h in RPMI. PBMCs were isolated by density gradient centrifugation and cultured under identical conditions. All conditions were triplicate. After incubation, samples were centrifuged (18,000 × g, 5 min), and supernatants collected. Cytokines and inter-ferons were quantified in duplicate using multiplex ELISA (Quansys Biosciences) according to the standardized protocol from the NCI’s NCL Assay Cascade described in detail earlier [26].

### 2.9. Animal studies

#### 2.9.1. Tumor model and treatment

All animal procedures were approved by the Institutional Animal Care and Use Committee at Wake Forest University Health Sciences. C57BL/6 mice were injected via the portal vein with 2 × 10^5^ MC38 cells. Tumor progression was monitored using IVIS Lumina III imaging. After tumor establishment, mice received CF10 (dose matched to 100 mg/kg 5-FU by A260), 5-FU (100 mg/kg), or vehicle control intravenously on day 0 and day 3. Mice were euthanized 3 days after the first dose and 7 days after the second dose for tissue collection.

#### 2.9.2. Spleen collection, splenocyte preparation, and T-cell analysis

Spleens were harvested, mechanically dissociated through 70 μm strainers, and subjected to RBC lysis [28]. Splenocytes were cultured with FBS and IL-2 for T-cell activation [29]. After stimulation, cells were stained for CD3, CD4, and CD8 (surface) and IFN-γ (intracellular) using fixation/permeabilization kits. Flow cytometry was performed on a BD FACSCanto II and analyzed with FlowJo.

#### 2.9.3. Dendritic cell vaccination in tumor-bearing mice

BMDCs were generated from C57BL/6 mice, pulsed for 24 h with supernatants from untreated (CTR-sup), CF10-treated (CF10-sup), or 5-FU-treated (5FU-sup) CRC cells, then washed. Tumor-bearing mice were immunized subcutaneously with 1 × 10^6^ pulsed BMDCs per mouse, three times at weekly intervals.

#### 2.9.4. Immunofluorescence and histological analysis

Tumors and livers were fixed, cryosection, and immunostained for CD4, CD8, γδ TCR, and MDSC markers (CD11b, Gr-1), followed by fluorescent secondary antibodies. Nuclei were counterstained with DAPI. Images were acquired using a Zeiss Axioplan 2 microscope and analyzed in ImageJ. Tumor sections were also processed for H&E staining. Metastatic tumor regions within liver tissue were identified using a serial-section approach. Tumor boundaries were first outlined on adjacent H&E-stained liver sections using characteristic features, including loss of hepatic cords and sinusoidal architecture, increased nuclear density, and the presence of compact tumor nests. These boundaries were then transferred to the corresponding IF sections using liver-specific anatomical landmarks such as portal triads, central veins, and lobular organization. Only viable tumor regions were included, while necrotic areas, large vessels, ducts, and artifacts were excluded. ROIs were manually delineated prior to quantifying CD3^+^, CD4^+^, and CD8^+^ T-cell infiltration.

### 2.10. Statistical analysis

Data were analyzed using GraphPad Prism v10.4.1. Tests included unpaired two-tailed Student’s *t*-test, one-way or two-way ANOVA, and log-rank (Mantel–Cox) test. Results are mean ± SD; significance defined as *p* < 0.05. Detailed methods and p-values are in figure legends.

## 3. Results

### 3.1. CF10 induces hallmarks of ICD in CRC cells in vitro

**Fig. 1.**
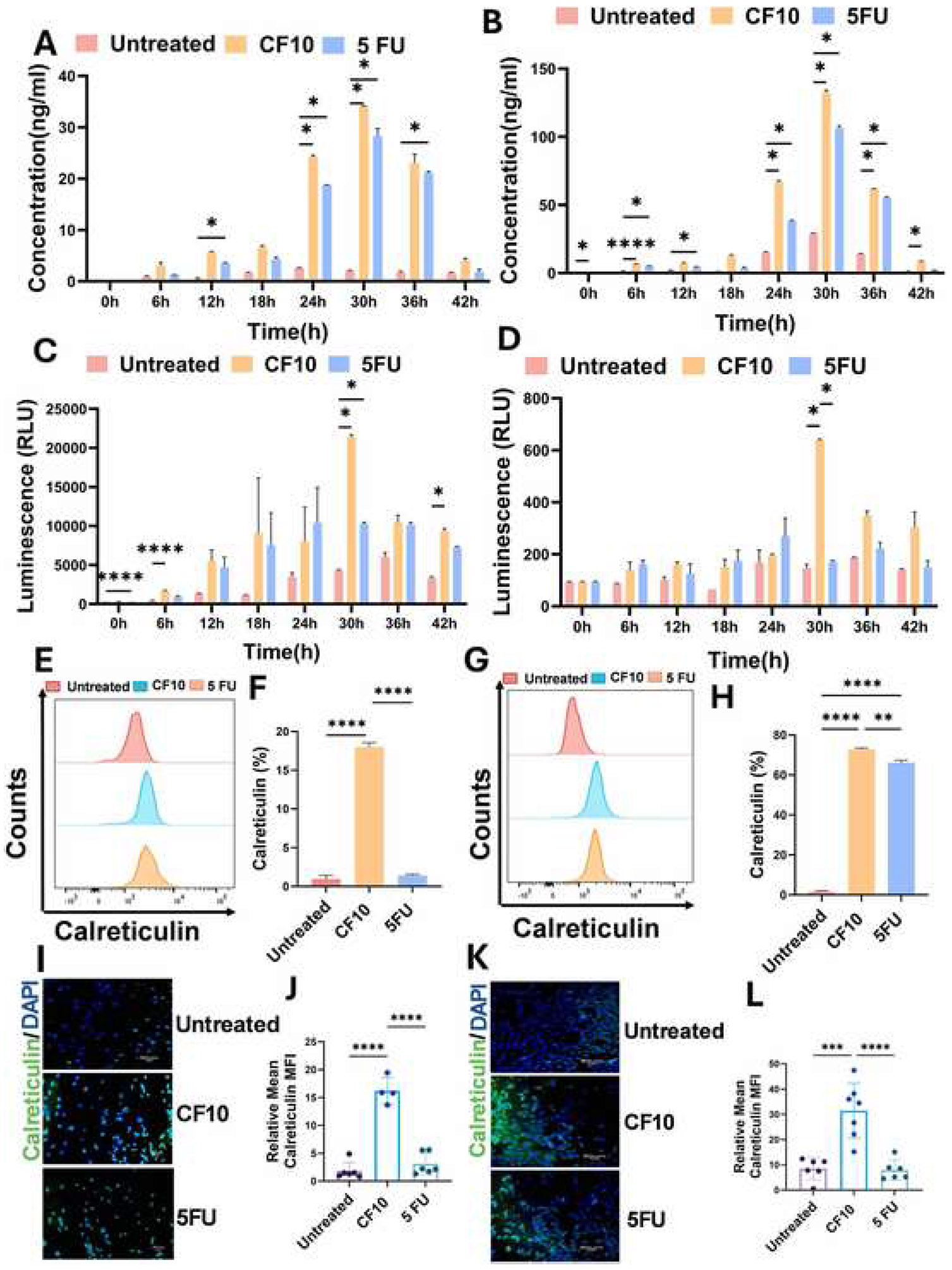
Induction of immunogenic cell death (ICD) markers in colorectal cancer (CRC) cells following treatment. MC38 (A, C) and HCT116 (B, D) cells were seeded at 3 × 10^5^ cells/mL and treated with either 1 μM CF10 or 10 μM 5-fluorouracil (5-FU) to deliver equivalent FP content. Culture supernatants were collected at the indicated time points (0–42 h) for analysis of ICD markers. (A) and (B), Release of high mobility group box 1 (HMGB1) and (C) and (D) extracellular ATP (eATP) levels. Data are shown as mean ± SD (n = 3); statistical significance was determined by two-way ANOVA (*, p < 0.05; **, p < 0.01; ***, p < 0.001; ****, p < 0.0001). Surface exposure of calreticulin (CRT) was assessed by flow cytometry in (E), MC38 and (G), HCT116 cells (5 × 10^5^ cells/mL) at 30 h post-treatment, with quantification shown in (F) and (H) respectively. CRT exposure was also visualized by fluorescence microscopy in (I) MC38 and (K) HCT116 cells, with corresponding quantification in (J) and (L). Data are shown as mean ± SD (n = 3); statistical significance was determined by one-way ANOVA (**, p < 0.01; ***, p < 0.001; ****, p < 0.0001).

Recent studies demonstrated significantly improved antitumor activity for CF10 and CF10/LV relative to 5-FU and 5-FU/LV in syngeneic, orthotopic rat [15] and mouse [16]models of CRC liver metastasis. To determine if the improved antitumor activity of CF10 results from activated antitumor immunity through induction of ICD we determined three accepted biomarkers of ICD - HMGB1 and extracellular ATP (eATP) secretion and calreticulin (CRT) surface expression [19]. Human (HCT-116) and mouse (MC38) CRC cells were treated with CF10 (1 uM) or 5-FU (10 uM) to deliver equivalent FP content. Both 5-FU and CF10 induced these three hallmarks of ICD in MC38 cells (Fig. 1A, C, E, F, I, J) and HCT-116 cells (Fig. 1B, D, G, H, K, L). The time course of HMGB1 and eATP secretion was similar for CF10 and 5-FU in both cell lines with maximum value at 30 h. CF10 (1 uM) induced significantly higher levels of HMGB1 and eATP relative to equivalent 5-FU (10 uM) at all timepoints. CF10 also induced higher levels of CRT surface expression than 5-FU at 30 h in MC38 (Fig. 1E and F) and HCT-116 (Fig. 1G and H) cells through flow cytometry. Immunofluorescence detection of surface calreticulin confirmed CF10 increased surface exposure significantly relative to untreated and 5-FU-treated MC38 (Fig. 1I and J) and HCT-116 cells (Fig. 1 K and L).

### 3.2. CF10 induces DNA damage, TOP1 cleavage complex formation, and stress signaling in colorectal cancer cells

**Fig. 2.**
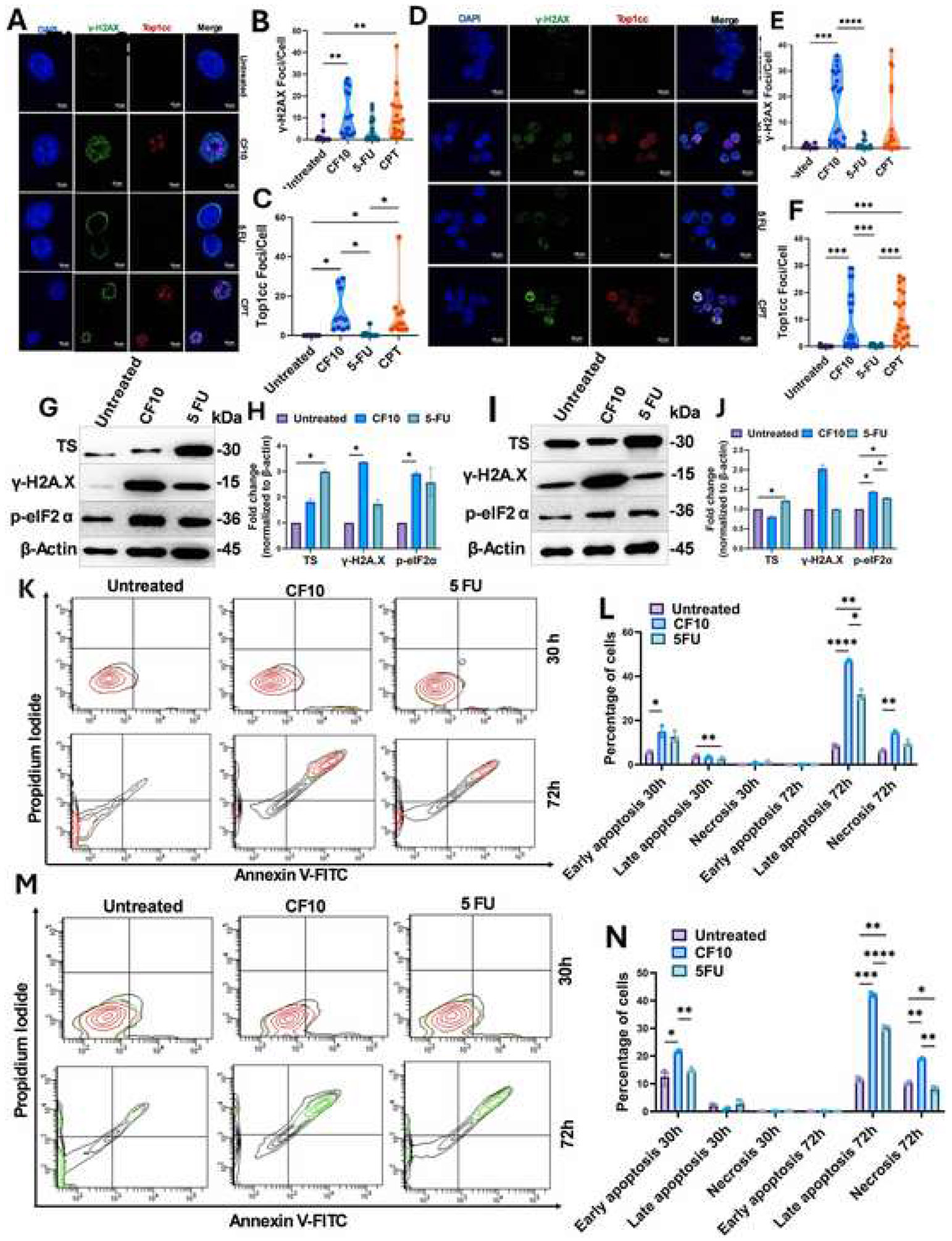
Mechanistic characterization of CF10 and 5-FU showing DNA damage, stress pathway activation, and apoptosis at 30 h. (A) Representative immunofluorescence images of MC38 cells treated for 30 h with 1 μM CF10, 10 μM 5-fluorouracil (5-FU) (equivalent fluoropyrimidine content), or 1 μM camptothecin (CPT; positive control), showing nuclear γ-H2AX and TOP1cc staining. Scale bars: 10 μm. (B, C) Quantification of γ-H2AX (B) and TOP1cc (C) foci per nucleus in MC38 cells. (D) Representative immunofluorescence images of HCT116 cells treated under the same conditions, showing γ-H2AX and TOP1cc staining. Scale bars: 20 μm. (E,F) Quantification of γ-H2AX (E) and TOP1cc (F) foci per nucleus in HCT116 cells. (G) Western blot analysis of MC38 lysates collected 30 h post-treatment, showing expression of thymidylate synthase (TS), γ-H2AX, and phospho-eIF2α, with β-actin as loading control. (H) Densitometric quantification of MC38 protein levels expressed as fold-change relative to untreated control and normalized to β-actin. (I,J) Western blot analysis (I) and fold-change quantification (J) of the same markers in HCT116 cells. (K) Annexin V–FITC/PI flow-cytometry plots of MC38 cells treated for 30 h or 72 h with CF10 or 5-FU, showing early apoptosis, late apoptosis, and necrosis. (L) Quantification of apoptotic subpopulations in MC38 cells. (M,N) Corresponding flow-cytometry analysis (M) and quantification (N) for HCT116 cells. Statistical analyses were performed using one-way ANOVA with appropriate post hoc tests; data are presented as mean ± SD; *p < 0.05, **p < 0.01, ***p < 0.001, **p < 0.0001.

CF10 strongly increased Top1cc and γ-H2AX foci in MC38 cells (Fig. 2A-C) compared with untreated and 5-FU controls, indicating enhanced Topoisomerase I cleavage complexes and DNA damage; CPT served as a positive control. In HCT116 cells (Fig. 2D-F), CF10 elicited the highest γ-H2AX response and robust Top1cc formation, surpassing CPT for DNA damage. Western blot analysis confirmed CF10-induced thymidylate synthase (TS) ternary complex formation and stronger DNA damage/stress signaling than 5-FU. In MC38 cells, CF10 increased TS (~1.8-fold), γ-H2AX (~3.3-fold), and p-eIF2α (~2.9-fold), versus smaller changes with 5-FU (Fig. 2G and H). In HCT116 cells. Flow cytometry showed CF10 promoted early apoptosis more effectively than 5-FU at 30 h (MC38: ~15% vs. 12.6%; HCT116: ~22% vs. 15%) (Fig. 2K and L). By 72 h, CF10 induced substantial late apoptosis (MC38: ~47%; HCT116: ~45%) compared with 5-FU (~32% and ~30%), with necrosis remaining low (Fig. 2M and N). Early apoptosis, accompanied by ER stress and surface calreticulin exposure, is consistent with an ICD-favorable phenotype. Collectively, these findings demonstrate that CF10 promotes DNA damage, replication stress, and apoptosis while activating pathways associated with immunogenic cell death, supporting its potential to stimulate adaptive antitumor immunity.

### 3.3. Supernatants from CF10-treated CRC cells promote DC maturation and cytokine secretion

**Fig. 3.**
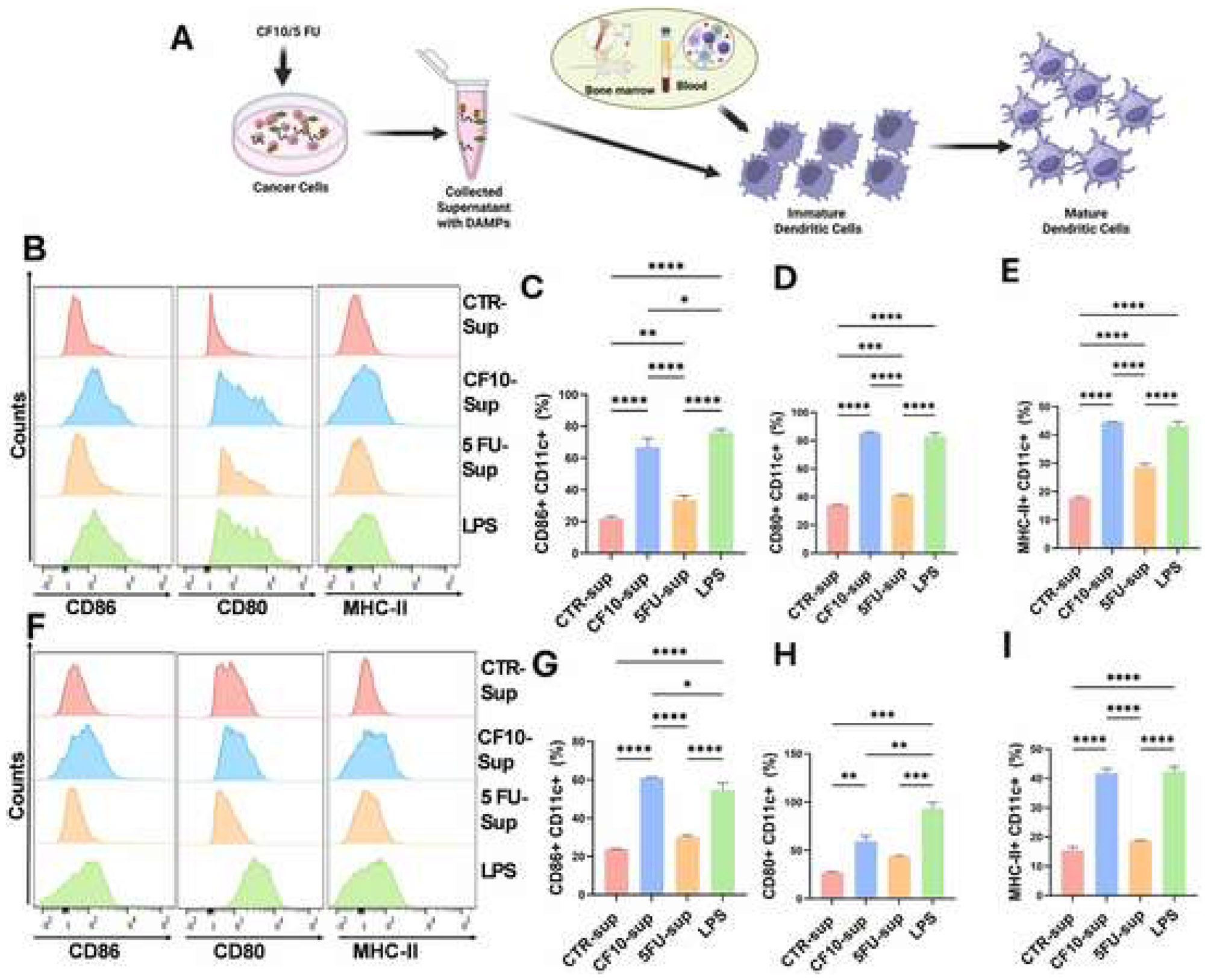
Supernatants from Chemically Stressed MC38 and HCT116 Cells Promote DC Maturation. (A) Schematic representation of the experimental workflow illustrating DC maturation induced by tumor cell–derived supernatants. (B) Mouse bone marrow–derived DCs (5 × 10^5^ cells/mL) cultured for 5 days were treated for 24 h with 100 μL/mL of cell-free supernatants obtained from MC38 cells treated with vehicle (CTR-sup), CF10 (CF10-sup), 5-fluorouracil (5-FU-sup), or with lipopolysaccharide (LPS; 100 ng/mL, positive control). Cells were analyzed by flow cytometry for surface expression of CD11c, CD80, CD86, and MHC class II (MHC-II). (C- E) Quantification of (C) CD80^+^ (D) CD86^+^ and (E) MHC-II^+^ cells within the CD11c^+^ murine DC population. (F) Human monocyte-derived DCs (5 × 10^5^ cells/mL) were similarly treated with supernatants from HCT-116 cells and analyzed for maturation marker expression. (G-I) Quantification of (G) CD80^+^, (H) CD86^+^ (I) and MHC-II^+^ cells within the CD11c^+^ human DC population. Data are presented as mean ± SD (n = 3). Statistical significance was determined by one-way ANOVA; * p < 0.05, **p < 0.01, ***p < 0.001, ****p < 0.0001.

Previous studies showed that supernatants from 5-FU-treated cancer cells can stimulate dendritic cell (DC) maturation [30]. To compare CF10 and 5-FU, MC38 cells were treated with CF10 (1 μM), 5-FU (10 μM), or left untreated; supernatants were added to mouse BMDC cultures (Fig. 3A). CF10-conditioned media significantly increased CD86, CD80, and MHC-II levels in CD11c^+^ DCs, whereas 5-FU elevated only MHC-II (Fig. 3B-E). Similar effects were observed in human monocyte-derived DCs added to CF10-treated HCT116 supernatants, while 5-FU had minimal effect (Fig. 3F-I). Bright-field microscopy confirmed morphological changes consistent with DC maturation (Supplementary Fig. S1), and CF10-treated LS174T supernatants increased surface DC markers and MHC-II (Supplementary Fig. S2).

**Fig. 4.**
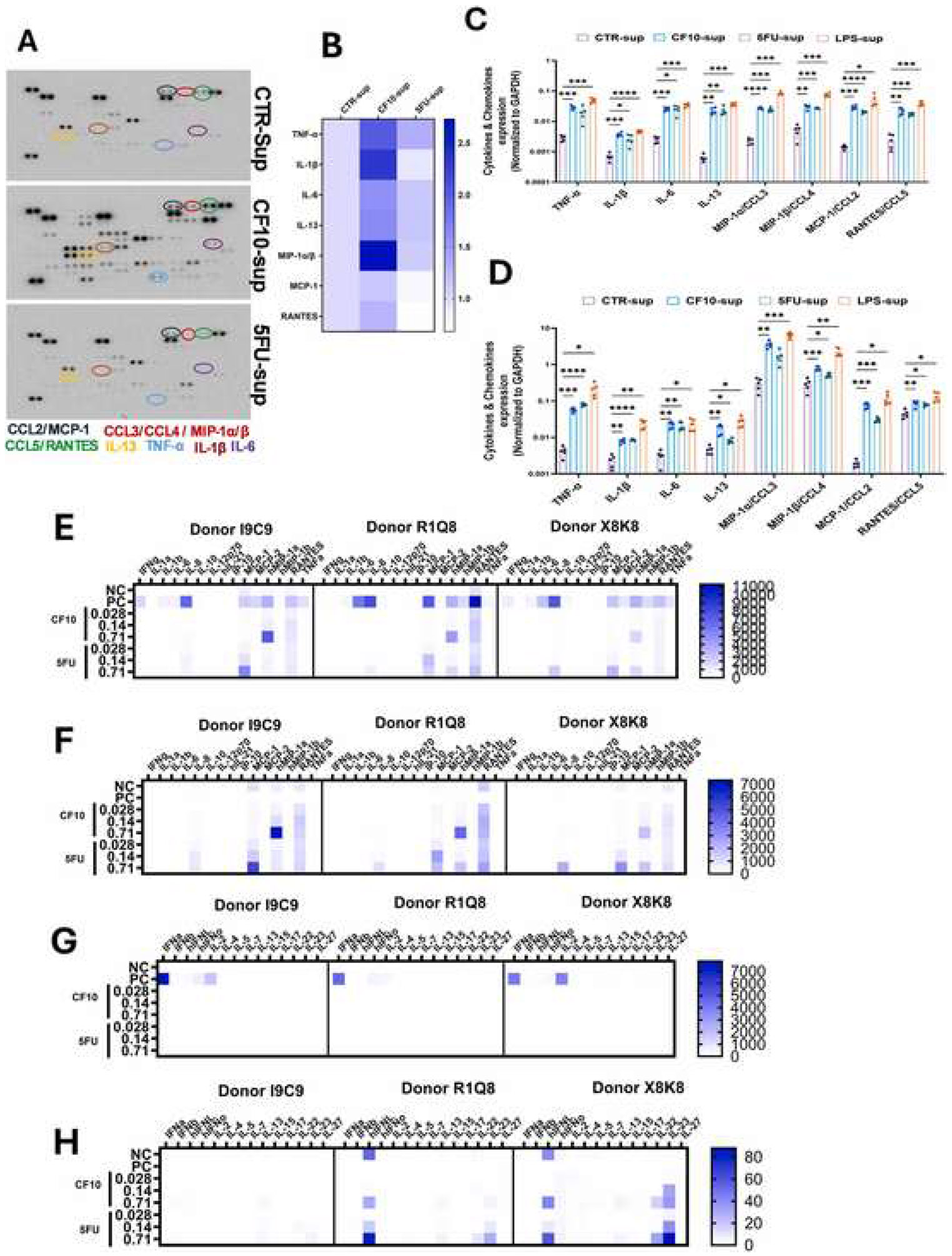
Chemokine and cytokine induction in DCs, whole blood, and PBMC cultures. (A) DCs: Mouse bone marrow-derived DCs (5 × 10^5^ cells/mL; 5-day culture) were stimulated for 24 h with 100 μL/mL of supernatants from untreated MC38 cells (CTR-sup), CF10-treated MC38 cells (CF10-sup), or 5-FU-treated MC38 cells (5-FU-sup). LPS (100 ng/mL) served as positive control. Chemokine and cytokine profiles were assessed using a cytokine array. (B) Spot intensities were quantified using ImageJ, normalized to positive control spots on the membrane, and expressed as fold change relative to the control group. The quantified data are visualized as a heatmap to illustrate differential cytokine expression patterns across treatment conditions. (C, D) mRNA expression: Selected chemokines and cytokines in DCs were measured by RT-qPCR after stimulation with MC38- (C) or HCT116-derived (D) supernatants. Values shown as mean ± SD (n = 5). Statistical significance by one-way ANOVA (*p < 0.05, **p < 0.01, ***p < 0.001, ****p < 0.0001). (E) Whole blood cultures: CF10 and 5-FU were compared. PBS was negative control; 20 ng/mL LPS plus 10 μg/mL PHA-M were positive controls. Cytokines were quantified by multiplex ELISA (mean ± SD, N = 3; analyzed in duplicate, CV < 20%). (F) Visualization: Positive control responses were masked for clarity in whole blood cultures. (G) PBMC cultures: Conditions matched whole blood experiments. Cytokines were quantified by multiplex ELISA (mean ± SD, N = 3; analyzed in duplicate, CV < 20%). (H) Visualization: Positive control responses were masked for PBMC cultures.

CF10 also enhanced DC cytokine/chemokine secretion. Mouse BMDCs pulsed to CF10 supernatants secreted higher CCL2, CCL3/4, CCL5, TNF-α, IL-1β, IL-6, and IL-13 than 5-FU or control (Fig. 4A,B), and RT-qPCR confirmed increased mRNA expression in both mouse BMDCs (Fig. 4C) and human DCs (Fig. 4D), with CF10 eliciting higher MIP-1α/CCL3 than 5-FU. In human whole blood, CF10 and 5-FU induced distinct chemokine profiles: CF10 resulted in physiologically significant (i.e., ≥ 2-fold above the baseline) increase in MIP1α and MIP-1β across all donor cultures, whereas 5-FU did so for MCP-1 and IL-8; these differences were also statistically significant for MIP1α, MCP-1 and IL-8 according to the two-way ANOVA Dunnett’s multiple comparisons test; both treatments resulted in comparable elevation of RANTES levels, though the elevated levels were neither physiologically nor statistically significant (Fig. 4E, F and Supplementary Figure S3A). In PBMC cultures, both compounds triggered low levels of IL-13 in all cultures, IL-22 in one donor (X8K8) culture, and IL-23 in two donor cultures (R1Q8 and X8K8); although these changes were not statistically significant by two-way ANOVA Dunnett’s multiple comparisons test, they were physiologically significant (i.e., ≥ 2-fold above the baseline) (Fig. 4F, G and Supplementary Figure S3B). These results indicate that CF10 is more effective than 5-FU at promoting CRC cell-mediated DC activation and cytokine/chemokine secretion in mouse and human immune cells.

### 3.4. CF10 stimulates a potent antitumor immune response contributing to antitumor activity

**Fig. 5.**
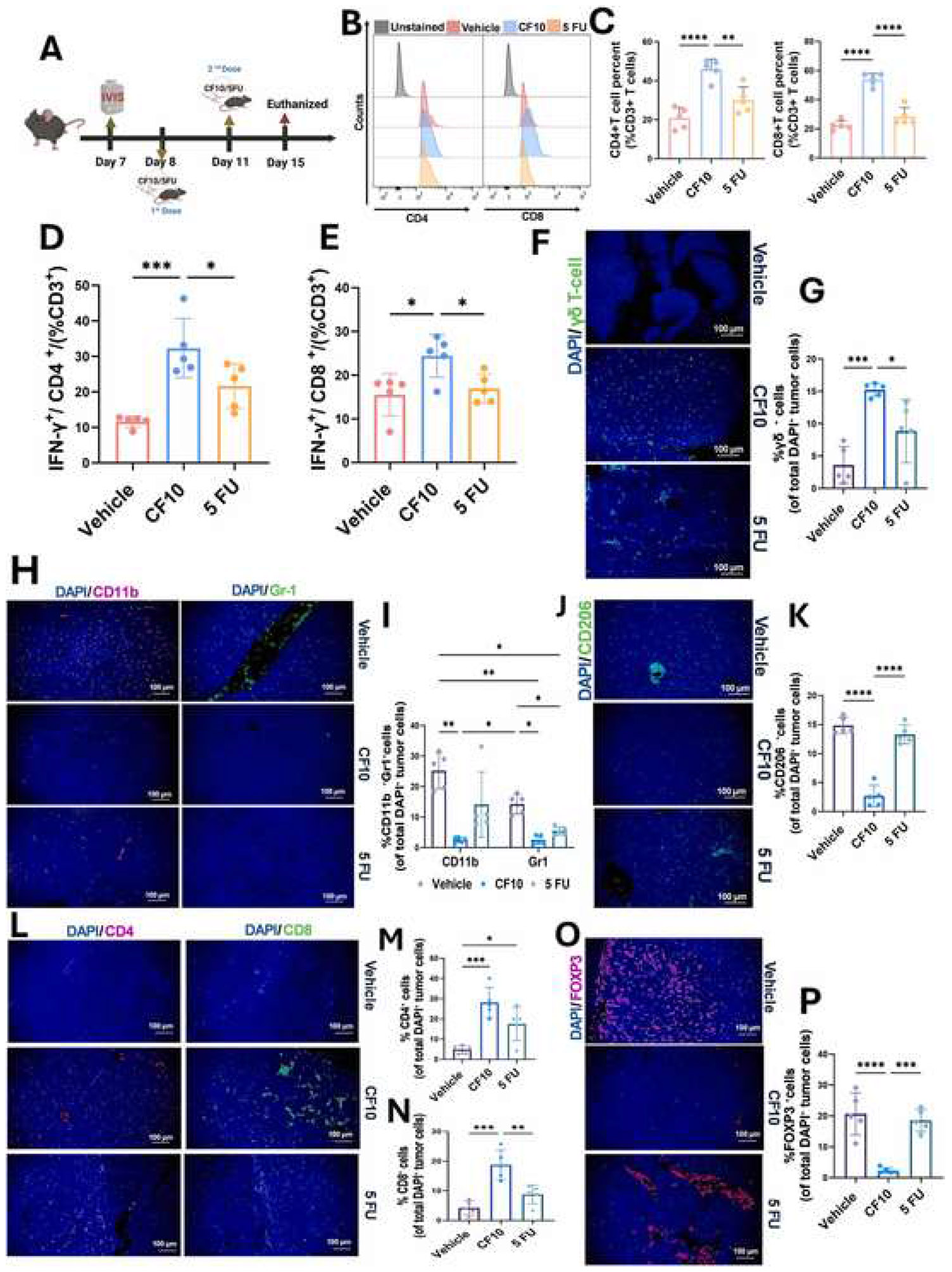
CF10 enhances systemic and hepatic T cell immunity in MC38 tumor-bearing mice. C57BL/6 mice were inoculated with 2 × 10^5^ MC38 tumor cells via portal vein injection. After tumor establishment, mice were treated intravenously with CF10 (equivalent to 100 mg/kg 5-FU based on equal A260 absorbance), 5-fluorouracil (5-FU, 100 mg/kg), or vehicle control. Treatments were administered on day 0 (initiation) and day 3. Mice were euthanized 7 days after the second dose, and tissues were collected for analysis. (A) Schematic of the experimental timeline. (B) Representative flow cytometry plots showing splenic CD4^+^ and CD8^+^ T cell populations at day 7 post-treatment (n = 5 mice per group). (C) Quantification of splenic CD4^+^ and CD8^+^ T cells, expressed as the percentage of CD4^+^ or CD8^+^ cells within the CD3^+^ T cell population. Statistical analysis was performed using one-way ANOVA. (D, E) Quantification of IFN-γ–producing CD4^+^ and CD8^+^ T cells in the spleen. Data represent the percentage of IFN-γ^+^ cells within each T-cell subset (CD4^+^ or CD8^+^) following stimulation. Statistical analysis was performed using one-way ANOVA. (F, G) Representative immunofluorescence images and quantification of hepatic γδ T cells. Data are expressed as the percentage of γδ T cells relative to total DAPI^+^ nuclei. Scale bar = 100 μm. (H, I) Representative immunofluorescence images and quantification of hepatic myeloid-derived suppressor cells (MDSCs; CD11b^+^Gr-1^+^), expressed as the percentage of MDSCs relative to total DAPI^+^ nuclei. Scale bar = 100 μm. Statistical analysis was performed using two-way ANOVA. (J, K) Immunofluorescence staining of liver sections showing CD206^+^ cells, with quantification expressed as the percentage of CD206^+^ cells relative to DAPI^+^ nuclei. Scale bar = 100 μm. Statistical analysis was performed using one-way ANOVA. (L–N) Immunofluorescence staining of liver sections showing CD4^+^ and CD8^+^ T cell infiltration across treatment groups, with quantification expressed as the percentage of CD4^+^ or CD8^+^ cells relative to DAPI^+^ nuclei. Scale bar = 100 μm. (O, P) Immunofluorescence staining of liver sections showing FOXP3^+^ cells, with quantification expressed as the percentage of FOXP3^+^ cells relative to DAPI^+^ nuclei. Scale bar = 100 μm. Statistical analysis was performed using one-way ANOVA. Data are presented as mean ± SD. Statistical significance is indicated as *p < 0.05, **p < 0.01, ***p < 0.001, ****p < 0.0001.

CF10 and CF10/LV previously reduced liver metastasis in mice and rats using sub-capsular CRC injections [31], a model that mimics tumor spread and pharmacological challenges [15,16]. To model metastatic seeding, MC38 cells were injected into the portal vein [21] of C57BL/6 mice and allowed to establish tumors for 7 days (Fig. 5A). Tumor formation was confirmed by IVIS imaging (n = 5/group; Supplementary Fig. S4A), followed by treatment on days 8 and 11 with CF10, 5-FU, or vehicle. 5-FU was given at 100 mg/kg (MTD), while CF10 was dosed to administer equivalent nucleotide content based on UV absorbance at 260 nm, or approximately 1/10 the molar content to account for CF10 being composed of 10 FdUMP nucleotides. All mice in each treatment group (n = 5) were euthanized 7 days after the first treatment and spleens were analyzed for T cells and IFN-γ (Fig. 5B-E) and livers were analyzed for immune cell infiltration (Fig. 5F-P). Livers were analyzed for evidence of visible tumor masses (Supplementary Fig. S4B, C) and spleens were analyzed for enlargement (Supplementary Fig. S4D, E). An identical study was performed except mice received treatment only one treatment on day 8 and mice were euthanized on day 11 (Supplementary Fig. S7). Spleens were analyzed for activated T cells (Supplementary Fig. S7B and C), and livers were considered for immune cell infiltration.

Quantification of splenic CD4^+^ and CD8^+^ T cells showed that both 5-FU and CF10 significantly increased these populations at days 3 and 7 post-treatment (Fig. 5B and C; Supplementary Fig. S7B and C), with CF10 producing a larger effect, particularly for CD8^+^ T cells at day 7. CF10-treated mice also had increased spleen size and weight (Supplementary Fig. S4D and E), whereas 5-FU slightly decreased spleen size. Flow cytometry revealed that CF10 significantly elevated IFN-γ levels in CD4^+^ and CD8^+^ T cells relative to vehicle and 5-FU, consistent with enhanced T-cell activation (Fig. 5D and E).

To assess whether the increase in peripheral T cells correlated with tumor infiltration, liver sections from mice collected at day 7 post-treatment were analyzed by immunofluorescence staining. Hepatic γδ T cells (γδ TCR; Fig. 5F, G), myeloid-derived suppressor cells (MDSCs; CD11b^+^Gr-1^+^; Fig. 5H, I), and the M2 macrophage marker CD206 (Fig. 5J, K) were evaluated to assess immune activation and immunosuppressive cell populations within the tumor microenvironment. In addition, infiltration of effector CD4^+^ and CD8^+^ T cells were examined (Fig. 5L-N), while regulatory T cells were identified by FOXP3 staining (Fig. 5O, P). Hematoxylin and eosin (H&E) staining confirmed substantial tumor burden in the vehicle and 5-FU groups (Supplementary Fig. S5). CF10 treatment markedly increased the infiltration of γδ, CD4^+^, and CD8^+^ T cells while reducing MDSCs, CD206^+^ immunosuppressive macrophages, and FOXP3^+^ regulatory T cells compared with the 5-FU and control groups, indicating reduced immunosuppression within the tumor microenvironment. Additional analysis of macrophages (F4/80) and natural killer cells (NK1.1) further characterized immune cell populations in the liver (Supplementary Fig. S6). Collectively, these findings indicate that CF10 treatment enhances infiltration of effector T cells and modulates immune cell populations within the liver tumor microenvironment, supporting a shift toward a more immunostimulatory, anti-tumor immune landscape, likely driven by the combined induction of immunogenic cell death (ICD), dendritic cell maturation, and T-cell recruitment. Liver sections from day 3 were stained for CD4^+^, CD8^+^, γδ T cells, and MDSCs (CD11b, Gr-1; Supplementary Fig. S7D-I). CF10 markedly increased infiltration of CD4^+^, CD8^+^, and γδ T cells while reducing MDSCs compared with control and 5-FU, indicating a sustained adaptive antitumor immune response in this CRC liver metastasis model.

### 3.5. In vivo vaccination with DCs stimulated with supernatants from CF10-treated MC38 cells inhibit tumor progression

**Fig. 6.**
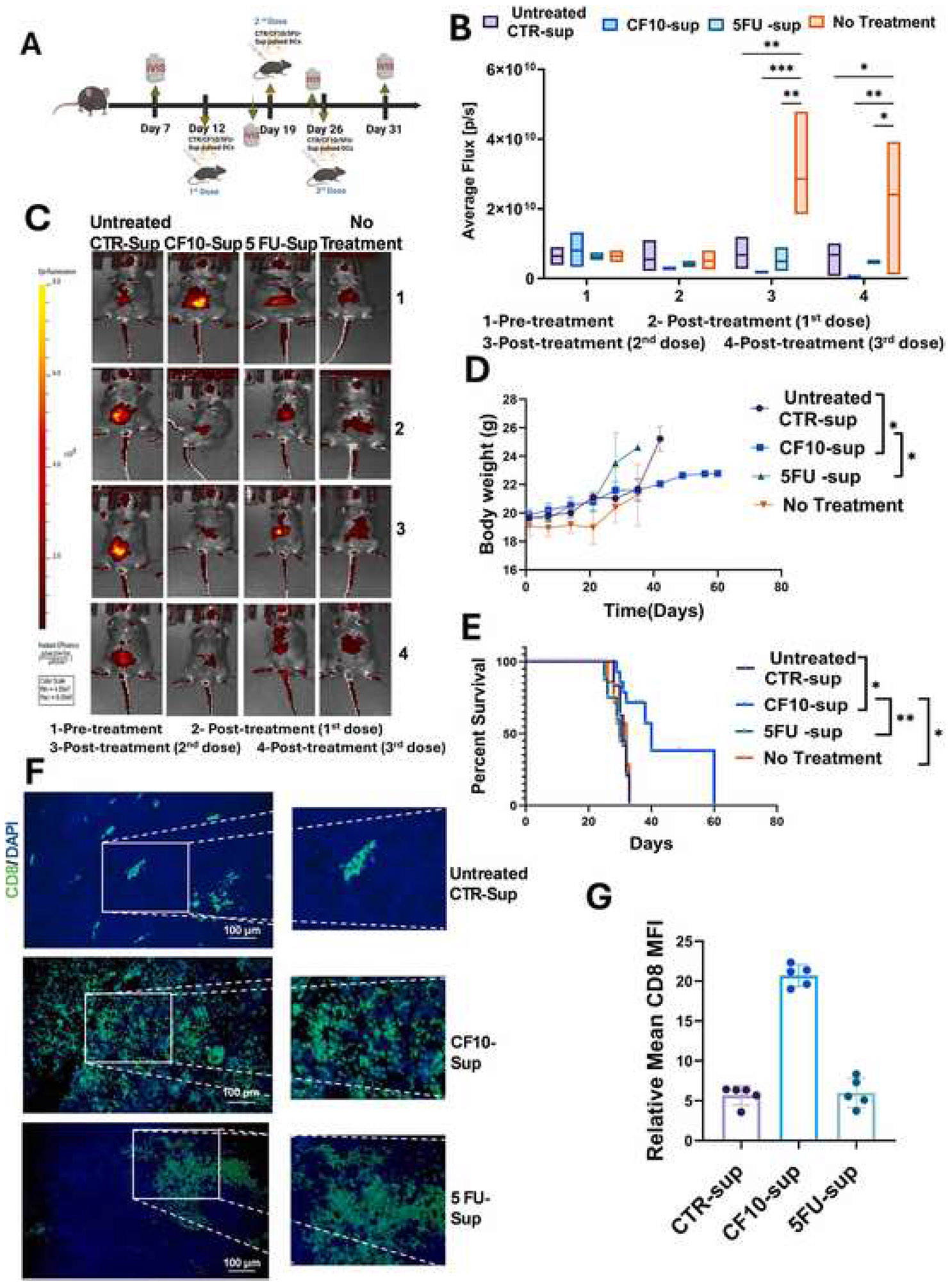
DCs Pulsed with Tumor Cell Supernatants Enhance Anti-Tumor Immunity in MC38 Liver Metastasis Model. C57BL/6 mice were challenged with 2 × 10^5^ MC38 tumor cells via portal vein injection, and tumor formation was confirmed using an *in vivo* imaging system (IVIS). Mice were subsequently immunized subcutaneously with DCs that were pulsed with supernatants derived from untreated MC38 cells (CTR-sup), CF10-treated cells (CF10-sup), or 5-fluorouracil-treated cells (5FU-sup). A group of tumor-bearing mice without immunization served as the control. (A) Schematic diagram of the experimental and treatment timeline. (B,C) Representative IVIS images showing tumor burden before and after DC immunization and quantification of IVIS signal intensity. (D) Body weight monitoring of mice throughout the study. (E) Kaplan–Meier survival curves following MC38 tumor challenge and DC-based immunization. Data are presented as mean ± SD (n = 6/group). (F, G) Representative images and quantification of CD8^+^ T-cell infiltration in tumor tissues at the study endpoint (immunofluorescence staining; scale bar = 100 μm). Statistical significance between groups was analyzed using one way and two-way ANOVA (p* < 0.05, p** < 0.01, ***p < 0.001, ****p < 0.0001).

To further establish CF10’s superior antitumor activity in the MC38:C57BL/6 orthotopic portal vein injection liver metastasis model, mice were vaccinated with DCs pulsed with supernatants from CF10-, 5-FU–treated, or untreated MC38 cells, alongside a no-treatment control. Tumor progression following portal vein injection of MC38 cells was evaluated (Fig. 6A). DCs were administered on days 12, 19, and 26, with tumor burden monitored thereafter. Untreated mice showed higher tumor flux than vaccinated groups, indicating modest antitumor activity of DCs pulsed with any MC38 supernatant. After the third dose, only CF10-pulsed DCs markedly suppressed tumor growth (Fig. 6B and C), with stable body weight (Fig. 6D). Kaplan–Meier analysis revealed a survival benefit for CF10-DC vaccination (Fig. 6E). Endpoint analysis showed increased intratumoral CD8^+^ T cells in CF10-DC–treated mice (Fig. 6F and G), supporting that CF10’s enhanced antitumor activity involves DC-mediated T-cell activation.

## 4. Discussion

Chemotherapy with 5-FU-based regimens (FOLFOX, FOLFIRI) remains central to the treatment of most patients with CRC liver metastases and results in improved overall survival but rarely enables long-term survival [2]. At present, use of immunotherapy for mCRC treatment is limited to patients with MSI-high tumors (~5% of mCRC patients) and CRC is considered an immune cold tumor with relatively few infiltrating T cells [4]. The effect of 5-FU treatment on a potential antitumor immune response is not established. However, at high doses 5-FU is used for myeloablation [9] and in clinical use 5-FU causes serious myelosuppression in some patients. Yet some pre-clinical studies demonstrate that 5-FU treatment contributes to an antitumor immune response in some contexts, for example by selective reduction in myeloid derived suppressor cell (MDSC) populations [32]. Overall, the lack of major improvements in overall survival even with optimized 5-FU-based chemotherapy regimens indicates a need to develop next generation fluoropyrimidine therapeutics that can both overcome acquired resistance to 5-FU/LV-based therapy [33] and stimulate a more potent antitumor immune response. CF10, a novel fluoropyrimidine polymer, outperforms 5-FU in CRC models by inducing TS inhibition, replication stress, and Top1cc stabilization, triggering ICD through DAMPs including extracellular ATP, HMGB1, and surface calreticulin.

Multiple preclinical studies have shown that CF10 provides superior therapeutic benefit compared with 5-FU in primary colon cancer [14] and CRC liver metastasis models, including syngeneic orthotopic models that preserve immune context [15,16]. CF10 is more potent than 5-FU against CRC cells and better tolerated in vivo, with lower systemic and hematopoietic toxicity. Its ability to induce immunogenic cell death (ICD) and antitumor immunity was unknown. CF10 triggered higher levels of canonical ICD markers—extracellular ATP, HMGB1, and surface calreticulin—in human (HCT116, LS174T) and murine (MC38) CRC cells, suggesting enhanced immune activation may underlie its therapeutic advantage.CF10’s increased cytotoxicity reflects dual targeting of thymidylate synthase and Topoisomerase 1, causing replication stress [34,35] and DNA damage (γ-H2AX). While the mechanistic link between cytotoxicity and ICD [36,37] remains unclear, our findings indicate that CF10’s combined TS/Top1 targeting and replication stress may underlie its superior ICD induction compared with 5-FU.

CF10-treated CRC supernatants strongly promoted dendritic cell (DC) maturation, increasing CD80, CD86, and MHC-II [20], and elicited a broader cytokine/chemokine profile than 5-FU [38], including TNF-α, IL-1β, IL-6, IL-13, and MIP-1α/β, MCP-1, and RANTES, supporting DC maturation and T-cell activation. In human whole blood, CF10 elevated MIP-1α/β, whereas 5-FU primarily increased MCP-1 and IL-8; RANTES was similar for both. PBMCs showed low IL-13, IL-22, and IL-23, indicating systemic immune modulation. Overall, CF10 induces stronger DC activation and a broader cytokine response than 5-FU, highlighting its immunomodulatory potential.

Consistent with CF10 inducing immunogenic cell death (ICD) and activating dendritic cells to stimulate T cell–mediated antitumor responses, CF10 treatment increased CD4^+^ and CD8^+^ T cells in the spleen and liver of mice with colorectal cancer (CRC) liver metastases formed via portal vein injection. Splenic T cells from CF10-treated mice secreted significantly more IFN-γ than those from 5-FU-treated mice, indicating enhanced activation. Intrahepatic γδ T cells were also elevated after CF10 therapy, a population known to exert cytotoxic effects, present antigens, and assist DC activation [39], suggesting a role in bridging innate and adaptive immunity. Importantly, CF10 significantly reduced immunosuppressive populations, including myeloid-derived suppressor cells (MDSCs), CD206^+^ M2 macrophages, and FOXP3^+^ regulatory T cells, alleviating inhibition of T-cell activation and promoting a supportive tumor microenvironment. Analysis of macrophages (F4/80) and natural killer cells (NK1.1) further indicated a shift toward enhanced innate immune surveillance. Collectively, CF10 promoted infiltration of effector CD4^+^, CD8^+^, and γδ T cells while reducing immunosuppressive populations, distinguishing it from 5-FU and highlighting its dual cytotoxic and immunomodulatory activity.

To investigate CF10-induced ICD in promoting adaptive antitumor immunity, we vaccinated mice with DCs pulsed ex vivo [30] with supernatants from CF10-treated MC38 cells in a portal vein CRC liver metastasis model. DCs pulsed with any MC38 supernatant modestly reduced tumor growth compared with non-pulsed DCs, indicating immune-stimulatory factors from tumor cells. However, CF10-conditioned DCs were significantly more effective than 5-FU or untreated supernatants, increasing intratumoral CD8^+^ T-cell infiltration, improving survival, and maintaining body weight, consistent with low toxicity. These results support that CF10 enhances antitumor immunity via DC maturation and T-cell activation. In addition to evaluating antitumor activity, establishing the systemic safety of CF10 is essential for its translational potential. The biochemical toxicity profile of CF10 has been comprehensively characterized in our prior work (14), where CF10 showed no evidence of hepatotoxicity or nephrotoxicity, in contrast to 5-FU, which produced marked elevations in ALT and AST. In the current study, we supplemented these findings with new histopathological analyses of liver, spleen, and kidney tissues from treated animals (Supplementary Fig. S10). Consistent with our earlier observations, CF10-treated mice exhibited normal tissue architecture without detectable inflammation or structural injury. Together, the integration of previously published biochemical markers with the newly generated histopathological data provides a robust and multi-level demonstration of the improved systemic safety of CF10 relative to 5-FU, supporting its further development as a more tolerable fluoropyrimidine therapeutic.

In conclusion, CF10 is a potent inducer of ICD in human and murine colorectal cancer, providing superior therapeutic benefit relative to 5-FU. In orthotopic liver metastasis models, CF10’s efficacy reflects both direct cytotoxicity and activation of adaptive immunity. Mechanistically, CF10 induces replication stress and Top1cc stabilization, causing thymidylate synthase inhibition, DNA damage (γ-H2AX), and integrated stress response activation (p-eIF2α). These stress signals converge with ICD pathways, leading to dendritic cell maturation, expansion and infiltration of effector T cells (CD4^+^, CD8^+^, γδ T cells), and reduction of immunosuppressive populations (MDSCs, FOXP3^+^ regulatory T cells, CD206^+^ macrophages), while enhancing NK1.1^+^ natural killer cells. ICD-associated DAMPs—calreticulin exposure, extracellular ATP, and HMGB1 release—further amplify immune engagement. CF10’s dual cytotoxic and immunogenic profile supports its clinical evaluation, including DAMP monitoring and combination strategies with immune checkpoint blockade or DNA damage response inhibitors to enhance durable adaptive antitumor immunity.

## Supplementary Material

Supplementary Material

This content has been supplied by the authors.

## Data Availability

Data will be made available on request.

## Appendix A. Supporting information

Supplementary data associated with this article can be found in the online version at doi:10.1016/j.biopha.2026.119399.

## Abbreviations:

5-FU: 5-Fluorouracil
mCRC: Metastatic Colorectal Cancer
CF10: Next-generation polymeric fluoropyrimidine CF10
ICD: Immunogenic Cell Death
eATP: Extracellular Adenosine Triphosphate
HMGB1: High Mobility Group Box 1
CRT: Calreticulin
DC: Dendritic Cell
BMDC: Bone Marrow–Derived Dendritic Cell
TS: Thymidylate Synthase
Top1: DNA Topoisomerase 1
DAMPs: Damage-Associated Molecular Patterns
ICB: Immune Checkpoint Blockade
FOLFOX: 5-FU, Leucovorin, Oxaliplatin regimen
FOLFIRI: 5-FU, Leucovorin, Irinotecan regimen
MDSC: Myeloid-Derived Suppressor Cell
PBMC: Peripheral Blood Mononuclear Cell
LPS: Lipopolysaccharide
IL: Interleukin
TNF-α: Tumor Necrosis Factor alpha
CCL: Chemokine C-C motif Ligand
MCP-1: Monocyte Chemoattractant Protein-1
MIP-1α/β: Macrophage Inflammatory Protein-1 alpha/beta
RANTES: Regulated upon Activation, Normal T Cell Expressed and Secreted
qRT-PCR: Quantitative Real-Time Polymerase Chain Reaction
PBS: Phosphate-Buffered Saline
FBS: Fetal Bovine Serum
MEM: Minimum Essential Medium
DMEM: Dulbecco’s Modified Eagle Medium
IVIS: In Vivo Imaging System
OCT: Optimal Cutting Temperature medium
H&E: Hematoxylin and Eosin
MFI: Mean Fluorescence Intensity
CTR: Control
Sup: Supernatant
RRID: Research Resource Identifier
