# Supplementary material for: Activation of an adaptive antitumor immune response by the polymeric fluoropyrimidine CF10 involves TS/Top1 dual targeting
Source: Biomed Pharmacother. Author manuscript; Available in PMC 2026 Jun 15. (PMC13266477; doi:10.1016/j.biopha.2026.119399)
Supplement: Supplementary Material [file NIHMS2172681-supplement-Supplementary_Material.pdf]

Supplementary Information

Activation of an adaptive antitumor immune response by the polymeric fluoropyrimidine CF10 involves TS/Top1 dual targeting

Akanksha Behl[a], Taylor M. Young[a], Xue Ma[b], Edward Cedrone[c], Marina A. Dobrovolskaia[c] , John F. Whitesides[d] and William H. Gmeiner[a*]

[a] Department of Cancer Biology, Wake Forest University School of Medicine, Winston-Salem, North Carolina, 27157, USA

[b] Department of Orthopedic Surgery and Rehabilitation, Medical Center Boulevard, Winston-Salem, North Carolina, 27157, USA

[c] Nanotechnology Characterization Laboratory, Cancer Research Technology Program, Frederick National Laboratory for Cancer Research sponsored by the National Cancer Institute, Frederick, Maryland, 21701 USA

[d] Microbiology & Immunology, Wake Forest University School of Medicine, Winston-Salem, North Carolina, 27157, USA

* Corresponding Author: William H. Gmeiner, Wake Forest University School of Medicine, Medical Center Blvd, Winston-Salem, NC 27055. Phone: 336-716-6216; Fax: 336-716-0255; Email: william.gmeiner@wfusm.org

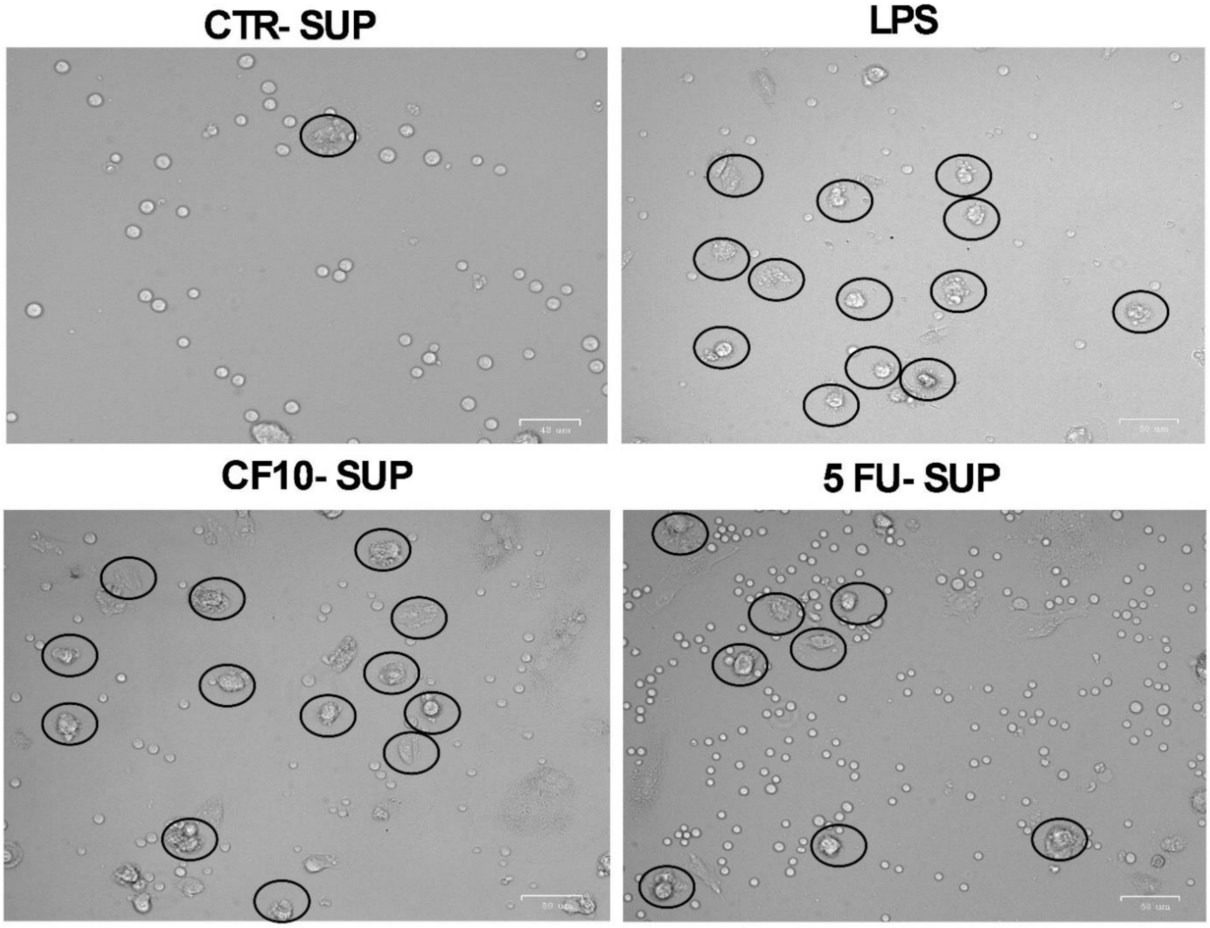

Supplementary Fig. 1. Maturation of human dendritic cells (DCs) induced by supernatants from chemically stressed HCT116 cells. Microscopic analysis of DCs treated with supernatants derived

from HCT116 cells subjected to various chemical treatments to assess morphological maturation. Scale bar 50= μm.

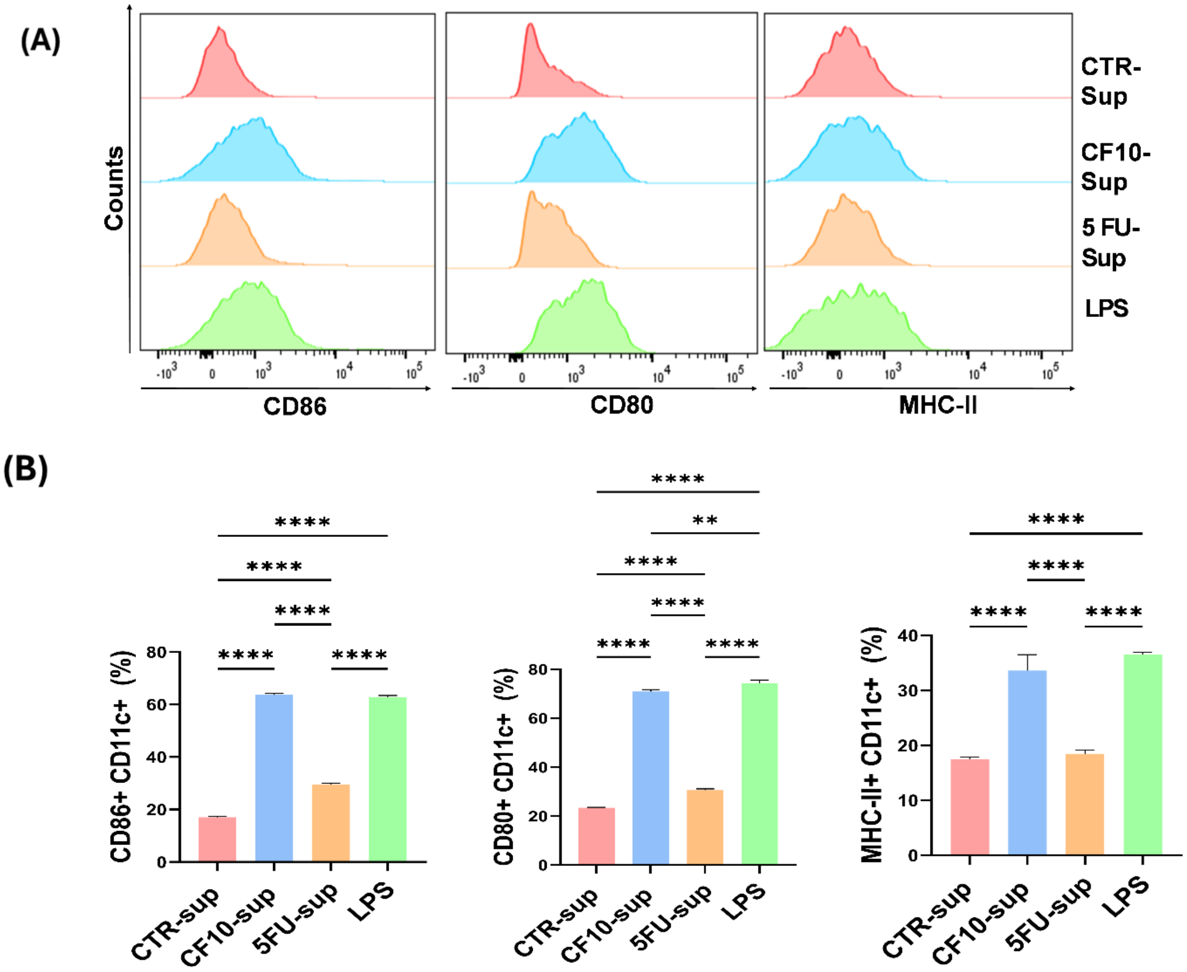

Supplementary Fig. 2. Functional maturation of human dendritic cells (DCs) induced by supernatants from chemically stressed LS174T cells. (A–B) Human monocyte-derived DCs (5×10^5 cells/mL; cultured for 5 days) were stimulated with supernatants from LS174T cells subjected to chemical stress. DC maturation was assessed by flow cytometry. Bar graphs show the frequencies of $CD86^+$, $CD80^+$, and $MHC\text{-}II^+$ cells among $CD11c^+$ DCs. Data are presented as mean ± SD (n = 3). Statistical analysis was performed using one-way ANOVA; $p < 0.01$, *$p < 0.001$, **$p < 0.0001$.

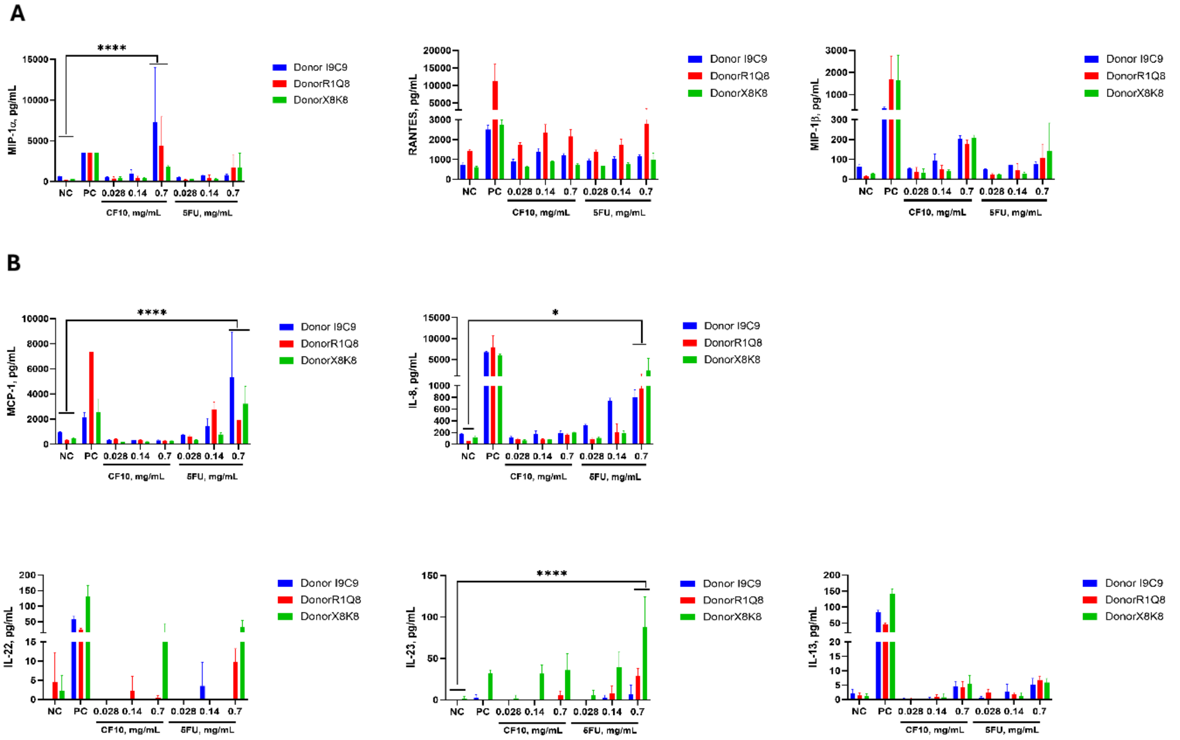

Supplementary Fig. 3 (A) Analysis of cytokines in whole blood cultures. Whole blood from healthy donor volunteers was exposed to negative control (NC), positive control (PC), CF10 or 5FU for 24 hours. PBS was used as NC, LPS, PHA-M and ODN2216 were used as PC. CF10 and 5FU were analyzed at a range of concentrations (0.0028 to 0.7 mg/mL). Culture supernatants were analyzed for the presence of 15 cytokines. The same data as in Figures 4D and 4E were replotted in this graph to show the induced cytokines in bar graph format. Cytokines that were not induced by CF10 or 5FU treatments are not plotted. (B) Analysis of cytokines in PBMC cultures. PBMCs from healthy donor volunteers were exposed to negative control (NC), positive control (PC), CF10 or 5FU for 24 hours. PBS was used as NC, LPS, PHA-M and ODN2216 were used as PC. CF10 and 5FU were analyzed at a range of concentrations (0.0028 to 0.7 mg/mL). Culture supernatants were analyzed for the presence of 14 cytokines. The same data as in Figures 4F and 4G were replotted in this graph to show the induced cytokines in bar graph format. Cytokines that were not

induced by CF10 or 5FU treatments are not plotted. Cytokine increase is considered physiologically significant when the levels in the treated sample are ≥ 2-fold above the baseline. Statistical significance was determined using two-way ANOVA Dunnett's multiple comparisons test across three donors. **** - p<0.0001, * - p<0.05).

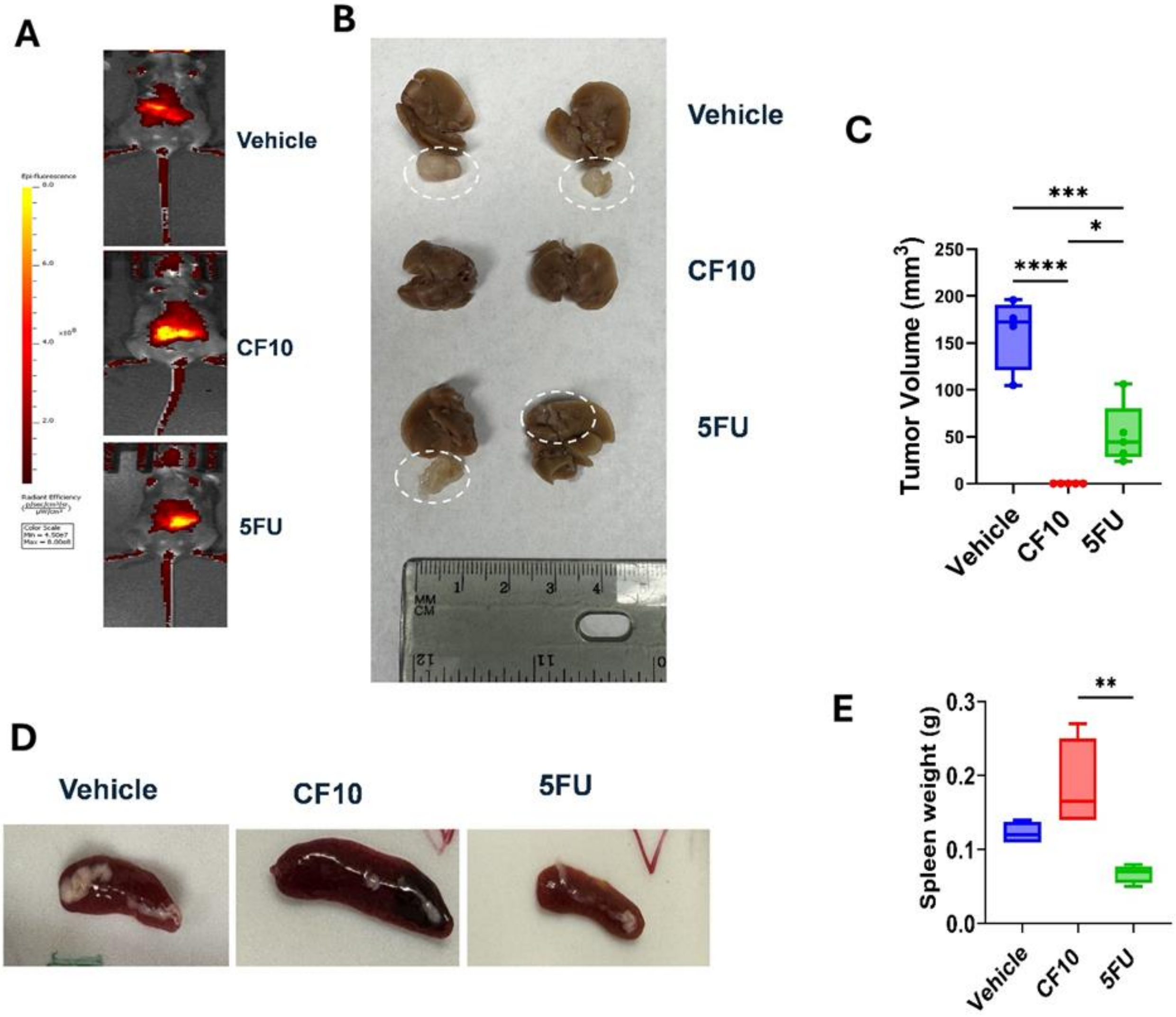

Supplementary Fig. 4. CF10 and 5-FU Effects on T Cell Responses, Tumor Progression, and Survival in MC38 Model.C57BL/6 mice were inoculated with 2×10^5 MC38 tumor cells via portal vein injection. After tumor establishment, mice were treated intravenously with CF10 (100 mg/kg), 5-fluorouracil (5-FU; 100 mg/kg), or vehicle control.(A) Representative IVIS images confirming tumor formation prior to treatment initiation.(B) Tumor burden assessed 7 days post-treatment.(C) Quantification of tumor volume after 7 days of treatment.(D) Representative images showing spleen size at day 7 post-treatment.(E) Quantification of spleen weight at day 7 post-treatment. Data are presented as mean ± SD (n = 5). Statistical significance was determined using one-way ANOVA; $p < 0.01$, *$p < 0.001$, **$p < 0.0001$.

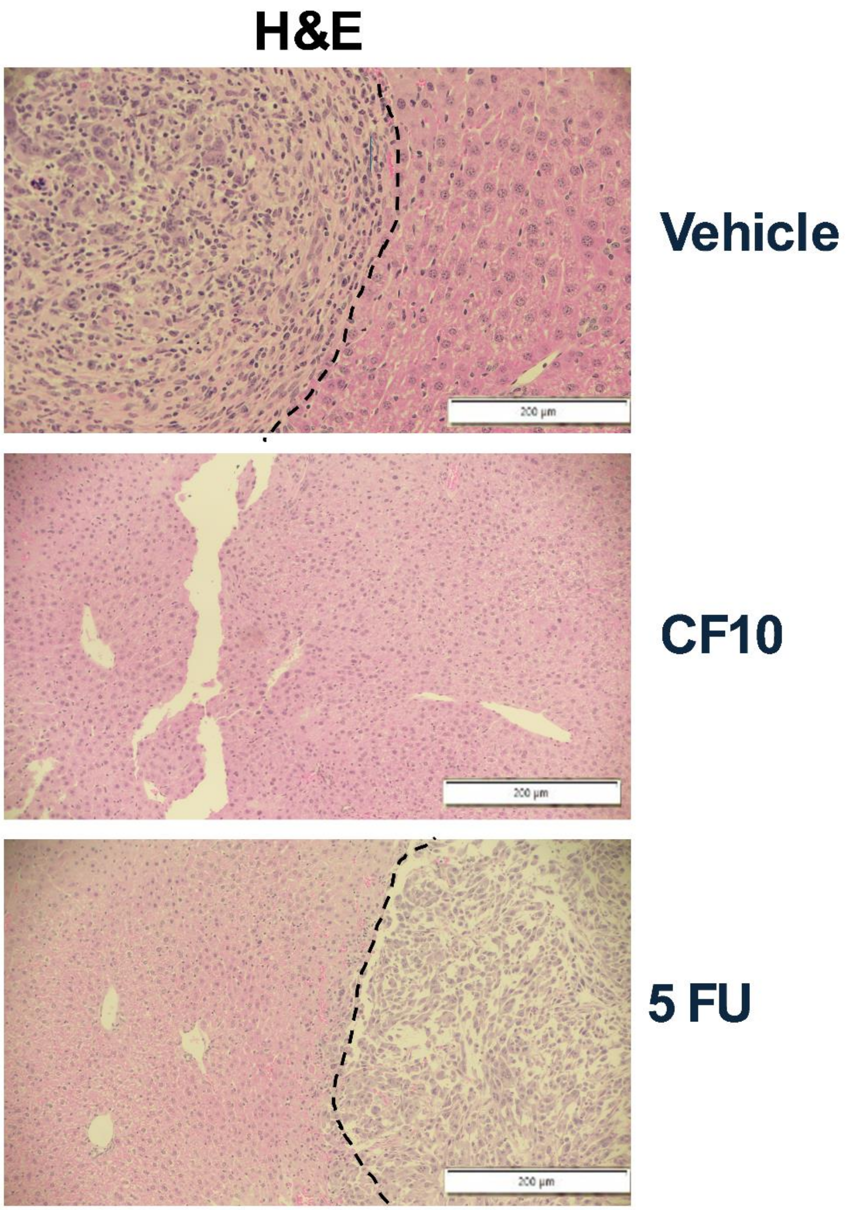

Supplementary Fig. 5. Representative hematoxylin and eosin (H&E)–stained sections of tumor tissue collected from mice treated with Vehicle, CF10, or 5-fluorouracil (5-FU). Images show tumor morphology and tumor–normal tissue interfaces at 200 µm scale. Dashed lines outline the boundary between tumor tissue and surrounding normal liver parenchyma for orientation.

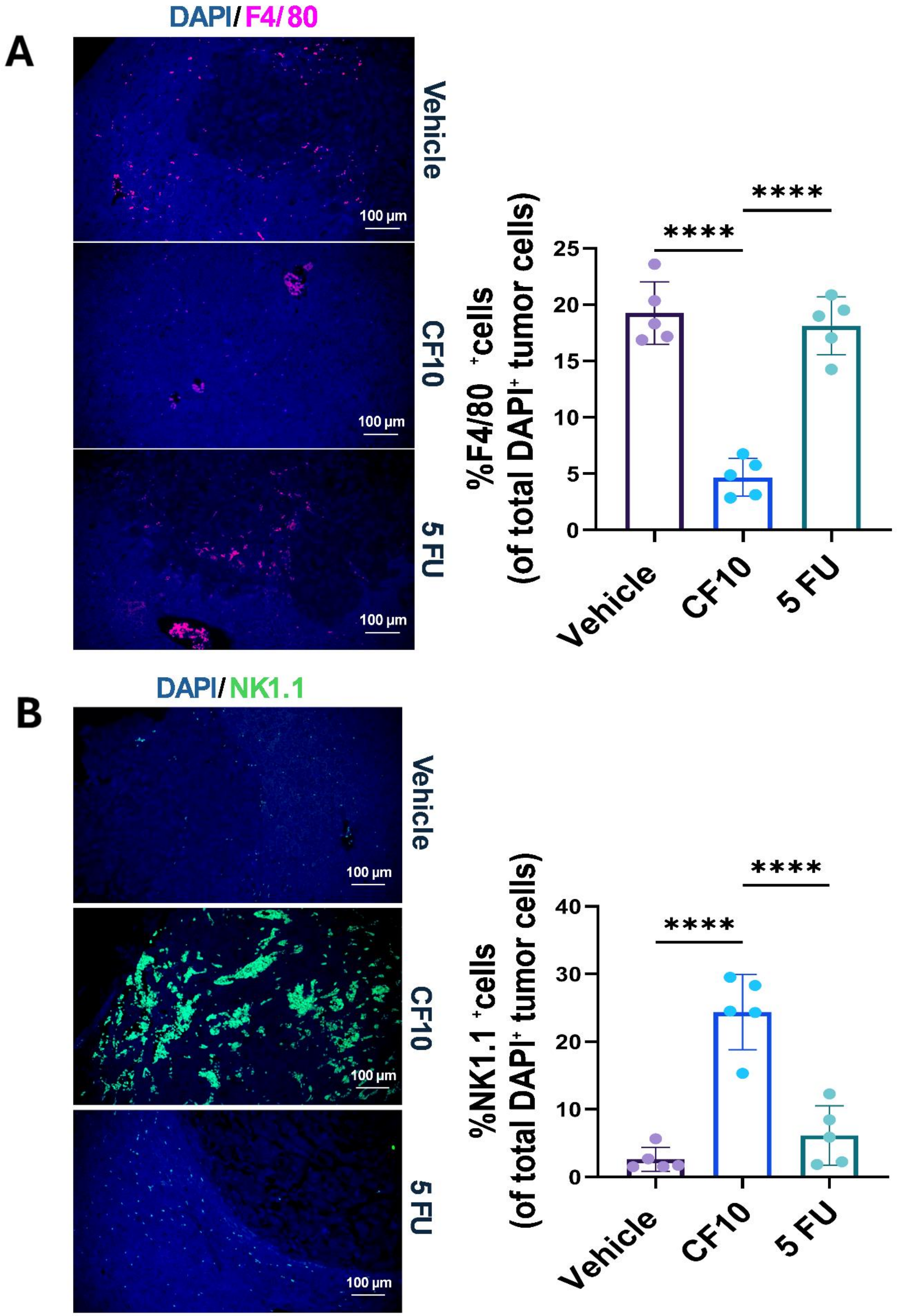
A
DAPI/F4/80
Vehicle
CF10
5 FU
100 µm
%F4/80 +cells (of total DAPI+ tumor cells)
0
5
10
15
20
25
****
****
Vehicle
CF10
5 FU
B
DAPI/NK1.1
%NK1.1 +cells (of total DAPI+ tumor cells)
0
10
20
30
40

73

Supplementary Fig. 6. Immunofluorescence analysis of hepatic immune cells. (A) Representative immunofluorescence images and quantification of F4/80^{+} cells, expressed as the percentage of F4/80^{+} cells relative to total DAPI^{+} nuclei. Scale bar = 100 μm. (B) Representative immunofluorescence images and quantification of NK1.1^{+} cells, expressed as the percentage of NK1.1^{+} cells relative to total DAPI^{+} nuclei. Scale bar = 100 μm. Quantification was performed on n = 5 mice per group. Data are presented as mean ± SD. Statistical significance was determined using one-way ANOVA; *$p < 0.05$, **$p < 0.01$, ***$p < 0.001$.

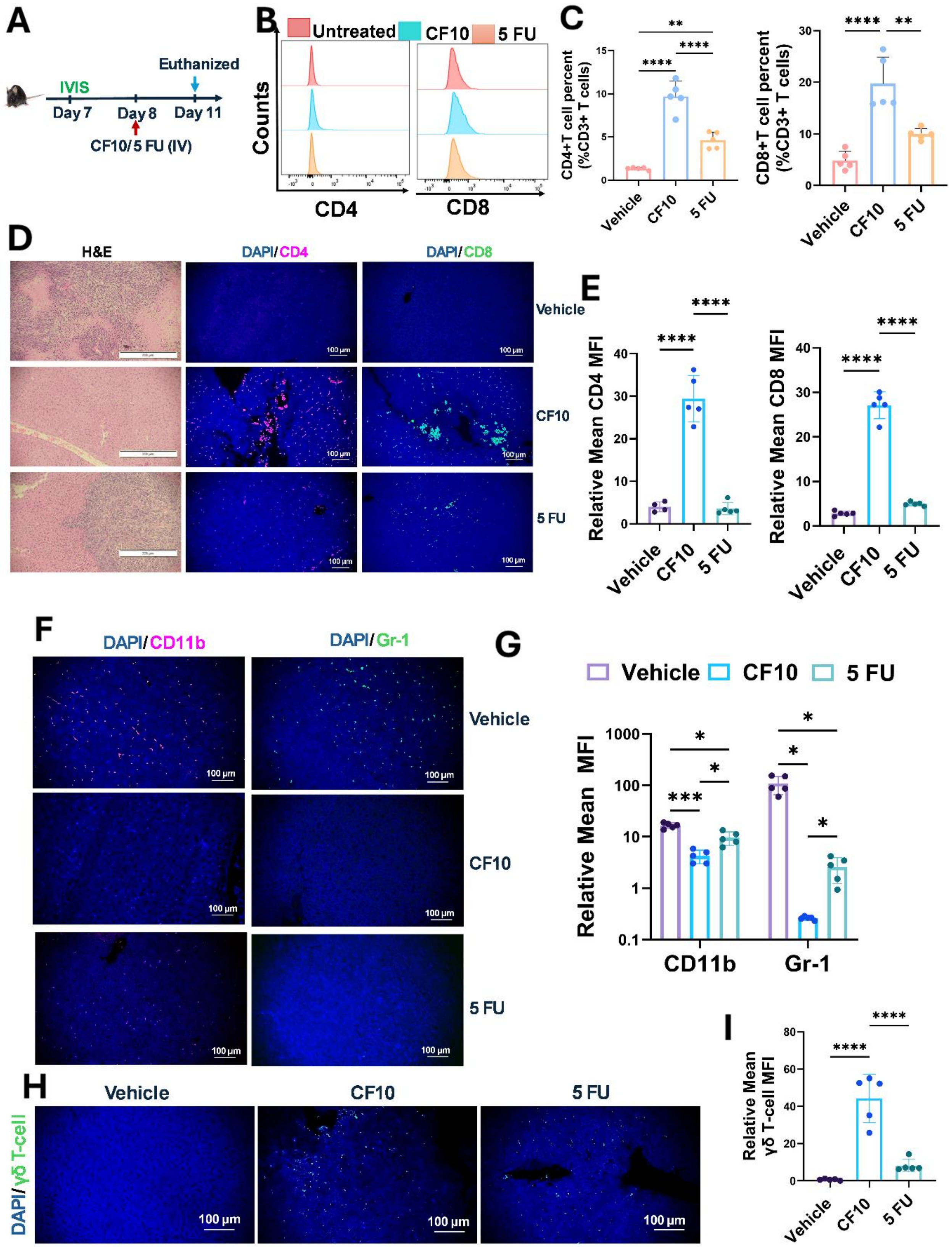
A
IVIS
Euthanized
Day 7
Day 8
Day 11
CF10/5 FU (IV)
B
Untreated
CF10
5 FU
Counts
CD4
CD8
C
CD4+T cell percent (%CD3+ T cells)
CD8+T cell percent (%CD3+ T cells)
Vehicle
CF10
5 FU
D
H&E
DAPI/CD4
DAPI/CD8
Vehicle
CF10
5 FU
100 µm
E
Relative Mean CD4 MFI
Relative Mean CD8 MFI
F
DAPI/CD11b
DAPI/Gr-1
G
Vehicle
CF10
5 FU
Relative Mean MFI
CD11b
Gr-1
H
DAPI/γδ T-cell
I
Relative Mean γδ T-cell MFI

82

Supplementary Fig. 7. CF10 treatment enhances systemic and intrahepatic T cell responses in MC38 tumor-bearing mice. C57BL/6 mice were inoculated with 2 × 10^5 MC38 tumor cells via portal vein injection. After tumor establishment, mice were treated intravenously with CF10 (matched to 100 mg/kg 5-FU based on equal A260 absorbance),5-fluorouracil (5-FU, 100 mg/kg), or vehicle control. Three days after the first treatment, mice were euthanized and various organs were collected for analysis.(A) Schematic representation of the experimental timeline.(B) Representative flow cytometry plots showing splenic $CD4^+$ and $CD8^+$ T cell populations 3 days after treatment initiation (n = 5 mice per group).(C) Quantification of splenic $CD4^+$ and $CD8^+$ T cells at day 3 post-treatment. Statistical analysis was performed using one-way ANOVA. (D–E) Immunofluorescence staining of liver sections showing $CD4^+$ and $CD8^+$ T cell infiltration across treatment groups. Relative mean fluorescence intensity (MFI) was calculated by normalizing to the control group and plotted in the corresponding graphs. Scale bar = 100 μm. Statistical significance was determined using one-way ANOVA. (F–G) Representative images and quantification of hepatic myeloid-derived suppressor cells (MDSCs; $CD11b^+Gr\text{-}1^+$) at day 3 post-treatment. Scale bar = 100 μm. Statistical significance was determined using two-way ANOVA. (H–I) Representative immunofluorescence images and quantification of hepatic γδ T cells at day 3 post-treatment. Scale bar = 100 μm Data are presented as mean ± SD. Statistical significance was determined using one-way or two-way ANOVA, as indicated; $^{**}p < 0.01$, $^{***}p < 0.001$, $^{****}p < 0.0001$.

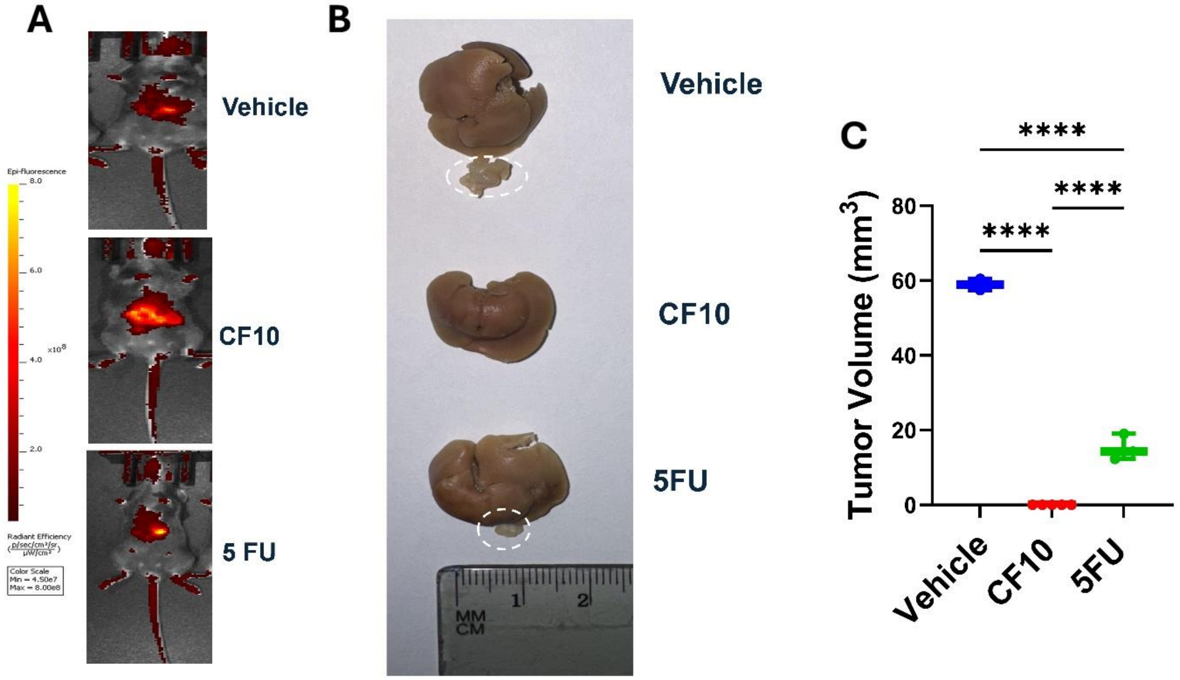

Supplementary Fig. 8. CF10 and 5-FU treatments suppress tumor growth in a portal vein-injected MC38 model. C57BL/6 mice were inoculated with 2×10^5 MC38 tumor cells via portal vein injection. After tumor establishment, mice were treated intravenously with CF10 (100 mg/kg), 5-fluorouracil (5-FU; 100 mg/kg), or vehicle control. (A) Representative IVIS images confirming tumor formation prior to treatment initiation. (B) Tumor burden assessed 3 days posttreatment. (C) Quantification of tumor volume after 3 days of treatment. Data are presented as mean ± SD ($n = 5$). Statistical significance was determined using one-way ANOVA; $p < 0.01$, *$p < 0.001$, **$p < 0.0001$.

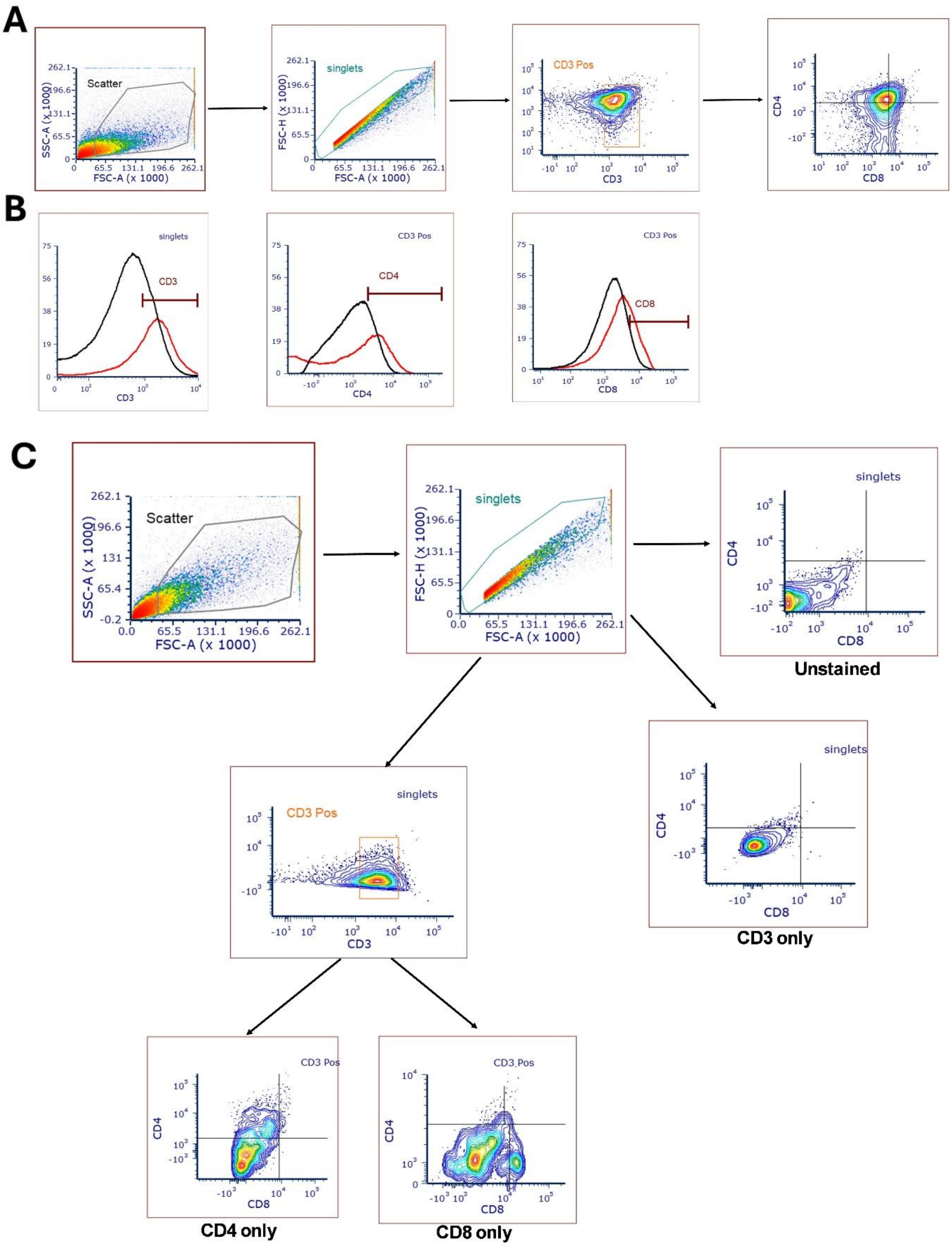
A
Scatter
SSC-A (x 1000)
FSC-A (x 1000)
singlets
FSC-H (x 1000)
CD3 Pos
CD3
CD4
CD8
B
singlets
CD3
CD3 Pos
CD4
CD8
C
Scatter
SSC-A (x 1000)
FSC-A (x 1000)
singlets
FSC-H (x 1000)
CD4
CD8
Unstained
CD3 only
CD3 Pos
CD3
CD4 only
CD8 only

Supplementary Fig. S9. Flow cytometry gating strategy and control staining used to define CD3$^+$, CD4$^+$, and CD8$^+$ T-cell populations (related to Fig. 5). (A) Primary gating workflow. Representative FSC–SSC density plot used to isolate lymphocytes, followed by singlet discrimination using FSC-A vs. FSC-H. CD3$^+$ events were subsequently gated from the singlet population, and downstream CD4 and CD8 expression were evaluated. Gating boundaries were determined using the control samples shown in Panels B and C.(B) Histogram overlays demonstrating marker-specific fluorescence separation. For each marker (CD3, CD4, CD8), single-stained samples (red) were overlaid with unstained controls (black) to show the shift in fluorescence intensity and to illustrate positive–negative separation. These controls were used to establish the thresholds for defining marker-positive populations. (C) Validation of gating boundaries using unstained and single-stained controls. Scatter and singlet gates from the experimental sample are shown on the left. The unstained control sample (top right) was used to define baseline autofluorescence and negative event boundaries for CD3, CD4, and CD8. Single-stained controls for CD3-only, CD4-only, and CD8-only (bottom row) allowed compensation and confirmation of true positive signals. Final CD4$^+$ and CD8$^+$ populations were gated based on these control-defined regions.

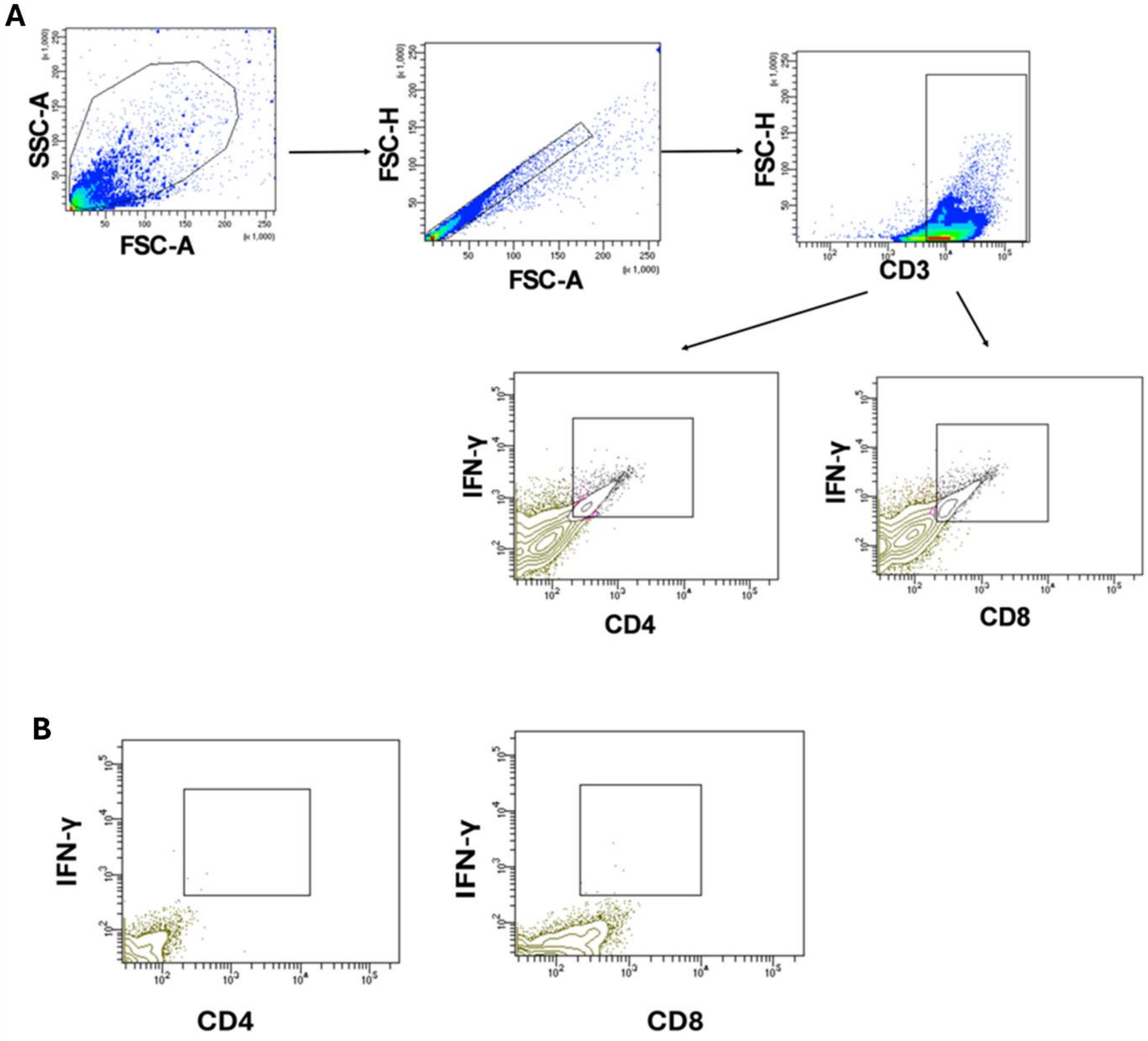

Supplementary Fig. S10. Gating strategy for identification of IFN-γ–producing $CD4^+$ and $CD8^+$ T cells. (A) Representative flow cytometry gating strategy. Lymphocytes were first selected based on FSC-A versus SSC-A, followed by singlet discrimination using FSC-A versus FSC-H. $CD3^+$ T cells were then identified and subsequently separated into $CD4^+$ and $CD8^+$ subsets. Intracellular IFN-γ production in each subset was quantified after stimulation. Boxes indicate the gates used to define IFN-γ–positive events. (B) Unstained controls for $CD4^+$ and $CD8^+$ T-cell populations. These samples were used to define background fluorescence and establish positivity thresholds for IFN-γ detection.

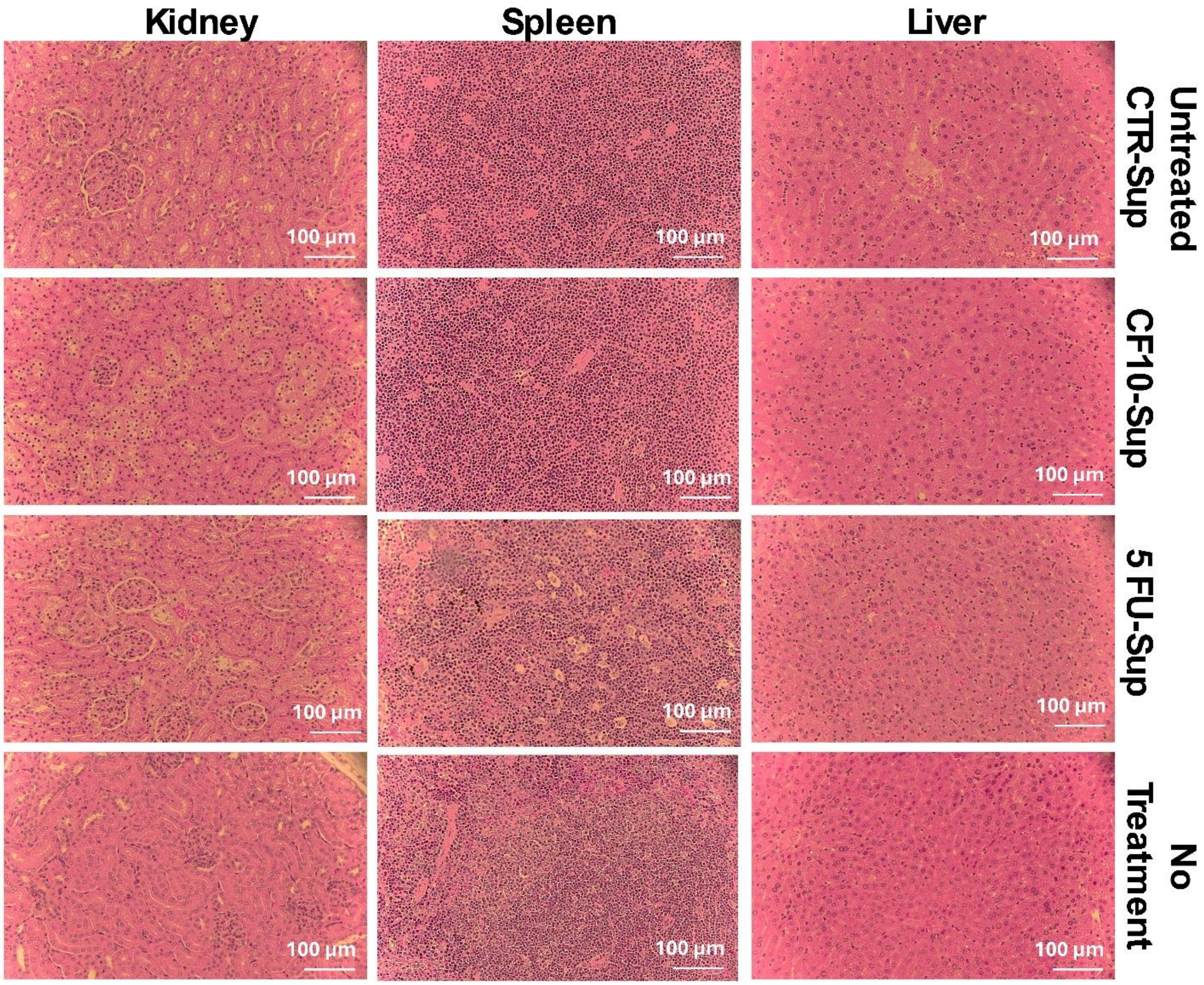

Supplementary Fig. 11. Representative H&E staining of liver, spleen, and kidney sections showing preserved tissue architecture. Scale bar = 100 µm.

Supplementary Table 1. List of reagents and their identifiers

Reagent/Resource	Reference or Source	Catalog Number/ RRID

**Experimental models**		
HCT116	ATCC	RRID: CVCL__0291
MC-38	ATCC	RRID: CVCL_ B288
LS174T	ATCC	RRID: CVCL_1384
C57BL/6 mouse	The Jackson Laboratory	RRID: IMSR_JAX:000664
**Antibodies**		
Calreticulin	Cell Signaling Technology	Cat# 12238, RRID: AB_2688013
PE anti-mouse CD80 Antibody	BioLegend	Cat# 104707, RRID: AB_313128
Alexa Fluor® 488 anti-mouse CD11c Antibody	BioLegend	Cat# 117313, RRID: AB_492849
APC anti-mouse CD86 Antibody	BioLegend	Cat# 105113, RRID: AB_313166
FITC anti-human CD11c Antibody	BioLegend	Cat# 337213, RRID: AB_1877174
APC anti-human CD86 Antibody	BioLegend	Cat# 305411, RRID: AB_493232
PE anti-human CD80 Antibody	BioLegend	Cat# 305207, RRID: AB_314503

APC anti-human HLA-DR Antibody	BioLegend	Cat# 327021, RRID: AB_2734310
PerCP anti-mouse I-A/I-E Antibody	BioLegend	Cat# 107623, RRID: AB_893586
FITC anti-mouse CD3 Antibody	BioLegend	Cat# 100203, RRID: AB_312660
APC anti-mouse CD4 Antibody	BioLegend	Cat# 305207, RRID: AB_314503
PE/Cyanine7 anti-mouse CD8a Antibody	BioLegend	Cat# 100721, RRID: AB_312760
Pacific Blue™ anti-mouse IFN-γ Antibody	BioLegend	Cat# 505817, RRID: AB_528922
Purified anti-mouse TCR γ/δ Antibody	BioLegend	Cat# 118101, RRID: AB_313826
Goat anti-Syrian Hamster IgG (H+L) Cross-Adsorbed Secondary Antibody, Alexa Fluor™ 488	Thermo Fisher Scientific	Cat# A-21110, RRID: AB_2535759

CD11b/ITGAM (E6E1M) Rabbit mAb	Cell Signaling Technology	Cat# 17800, RRID: AB_3665018
Ly-6G/Ly-6C (Gr-1) (RB6-8C5) Rat mAb (FITC Conjugate)	Cell Signaling Technology	Cat# 68590, RRID: AB_2936376
Anti-rabbit IgG (H+L), $F(ab')_2$ Fragment (Alexa Fluor® 488 Conjugate)	Cell Signaling Technology	Cat# 4412, RRID: AB_1904025
Anti-rabbit IgG (H+L), $F(ab')_2$ Fragment (Alexa Fluor® 647 Conjugate)	Cell Signaling Technology	Cat# 4414, RRID: AB_10693544
CD4 (D7D2Z) Rabbit mAb	Cell Signaling Technology	Cat# 25229, RRID: AB_2798898
CD8α (D4W2Z) XP® Rabbit mAb	Cell Signaling Technology	Cat# 98941, RRID: AB_2756376
Phospho-eIF2 alpha (Ser51) (D9G8) Rabbit Monoclonal Antibody	Cell Signaling Technology	Cat# 3398, RRID: AB_2096481
Thymidylate Synthase Antibody	Cell Signaling Technology	Cat# 3766, RRID: AB_2210584
Phospho-Histone H2A.X (Ser139) Antibody	Cell Signaling Technology	Cat# 2577, RRID: AB_2118010

Beta-Actin (13E5) Rabbit Monoclonal Antibody	Cell Signaling Technology	Cat# 4970, RRID: AB_2223172
Anti-Topoisomerase I-DNA Covalent Complexes Antibody	Millipore Sigma	Cat# MABE1084, RRID: AB_2756354
**Oligonucleotides and other sequence-based reagents**		
Primers are listed in Table ST2		
**Chemicals, Enzymes and other reagents**		
DMEM	Gibco	11995065
RPMI 1640	Gibco	11875093
FBS	Gibco	16000044
Penicillin-Streptomycin	Gibco	15140122
MEM	Gibco	11095080
McCoy's 5A Medium	Gibco	16600082
Recombinant Murine GMCSF	Peprotech	315-03
Recombinant human GMCSF	Peprotech	212-12
Lipopolysaccharides from *Escherichia coli* O55:B5	Sigma-Aldrich	L4524
Recombinant Murine IL-2	Peprotech	212-12
RBC Lysis Buffer(10X)	BioLegend	420301
Intracellular Staining Permeabilization Wash Buffer	BioLegend	421002
Human IL-4 Recombinant Protein	Peprotech	200-04

ProLong™ Gold Antifade Mountant	Thermo Fisher Scientific	P36934
Albumin, Bovine Serum	Sigma-Aldrich	12660
**Commercial assays**		
RNeasy Kits for RNA Isolation	Qiagen	74104
I Script CDNA synthesis kit	Bio-Rad	1708890
iTaq™ Universal SYBR® Green supermix	Bio-Rad	1725121
RealTime-Glo™ Extracellular ATP Assay	Promega	GA5010
Lumit® HMGB1 Human/Mouse Immunoassay	Promega	W6110
Proteome Profiler Mouse XL Cytokine Array	R& D Systems	ARY028
Alexa Fluor 488 /Annexin V & Dead Cell Apoptosis Kit	Invitrogen	V13241
**Drugs**		
CF10	ST Pharm	
Clinical-grade 5-fluorouracil (5-FU)	Baptist Hospital clinical pharmacy	
**Software**		

GraphPad Prism (V10.4.1)	http://www.graphpad.com/	(RRID:SCR_002798)
ImageJ	https://ij.imjoy.io/	(RRID:SCR_003070)
FlowJo (v10.8.1)	https://www.flowjo.com/	(RRID:SCR_008520)

Supplementary Table 2. RT PCR primers for cytokines and chemokines

Gene	Primer Sequence (5'-3')
TNF-α Human F	TCCTCAGCCTCTTCTCCTTC
TNF-α Human R	GATGATCTGACTGCCTGGG
TNF-α Mouse F	CGTGGAACTGGCAGAAGAG
TNF-α Mouse R	CCGAAGTTCAGTAGACAG
GAPDH Human F	CCTCAACTACATGGTTTACATGTT
GAPDH Human R	TTG ATT TTG GAG GGA TCT CGC
GAPDH Mouse F	CCCCTTCATTGACCTCAACTA
GAPDH Mouse R	GGCGGAGATGATGACCCTTT
IL6 Human F	AGACAGCCACTCACCTCTTCAG
IL6 Human R	TTCTGCCAGTGCCTCTTTGCTG
IL6 Mouse F	AATTTCCTCTGGTCTTCTGGAGT
IL6 Mouse R	ATCTCTCTGAAGGACTCTGGC
IL-1β Human F	GTGGCAATGAGGATGACTTGTTCT
IL-1β Human R	TGTAGTGGTGGTCGGAGATTCG
IL-1β Mouse F	TGCCACCTTTTGACAGTGATG

IL-1β Mouse R	AAGGTCCACGGGAAAGACAC
IL-13 Human F	AGCCCTGGAATCCCTGAA
IL-13 Human R	ACCTTGTGCGGGCAGAAC
IL-13 Mouse F	TGTAGCCCTGGATTCCCTG
IL-13 Mouse R	CTTGCGGTTACAGAGGCC A
MIP-1 α Human F	TGACACGCCGACCGCCT
MIP-1 α Human R	CCGGGCTTGGAGCACTG
MIP-1 α Mouse F	GGAGCTGACACCCCGAC
MIP-1 α Mouse R	GACACCTGGCTGGGAGC
MIP-1 β Human F	GGGCTCAGACCCTCCCA
MIP-1 β Human R	CTGGGAGCAGAGGCTGC
MIP-1 β Mouse F	TCTGACCCTCCCACTTCCT
MIP-1 β Mouse R	GGCTTGGAGCAAAGACTGC
MCP-1 Human F	CAATCAATGCCCCAGTCACC
MCP-1 Human R	CTTCTATAGCTCGCGAGC
MCP-1 Mouse F	GTTAACGCCCCACTCACCT
MCP-1 Mouse R	TTTGGGACACCTGCTGCTG
RANTES Human F	CTGCTTTGCCTACATTGCCC
RANTES Human R	TGGAGCACTTGCCACTGGT
RANTES Mouse F	CACCACTCCCTGCTGCTTT
RANTES Mouse R	TGCACTTGCTGCTGGTGTA
